# Biobased Waterborne Polyurethane-Urea/SWCNT Nanocomposites for Hydrophobic and Electrically Conductive Textile Coatings

**DOI:** 10.3390/polym13101624

**Published:** 2021-05-17

**Authors:** Amado Lacruz, Mireia Salvador, Miren Blanco, Karmele Vidal, Amaia M. Goitandia, Lenka Martinková, Martin Kyselka, Antxon Martínez de Ilarduya

**Affiliations:** 1 Color Center, S.A. Ptge. Marie Curie 3, Nau 6, 08223 Terrassa, Spain; msalvador@colorcenter.es; 2 Departament d’Enginyeria Química, Universitat Politècnica de Catalunya, ETSEIB, Diagonal 647, 08028 Barcelona, Spain; antxon.martinez.de.ilarduia@upc.edu; 3 Tekniker, Basque Research and Technology Alliance (BRTA), Surface Chemistry and Nanotechnology Unit, Iñaki Goenaga 5, 20600 Gipuzkoa, Spain; miren.blanco@tekniker.es (M.B.); karmele.vidal@tekniker.es (K.V.); amaia.martinez@tekniker.es (A.M.G.); 4 Inotex spol. s r.o, Stefanikova 1208, 54401 Dvur Kralove n.L., Czech Republic; martinkova@inotex.cz (L.M.); kyselka@inotex.cz (M.K.)

**Keywords:** waterproof, water-column, fluorine-free, bio-based, hydrophobic, electrically conductive textiles, nanocomposites, technical textiles, multifunctional fabrics, textile coatings

## Abstract

Waterborne polyurethane-urea dispersions (WPUD), which are based on 100% bio-based semi-crystalline polyester polyol and isophorone diisocyanate, have been successfully synthesized and doped with single-walled carbon nanotubes (SWCNT) to obtain a finishing agent that provides textiles with multifunctional properties. The chemical structure of WPUD has been characterized by Fourier-transform infrared spectroscopy (FTIR) and nuclear magnetic resonance (NMR). The thermal properties have been evaluated by differential scanning calorimetry (DSC), thermogravimetric analysis (TGA), and dynamic mechanical thermal analysis (DMTA). Mechanical properties have been studied by tensile stress–strain analysis. Moreover, the particle size, particle size distribution (PSD), and stability of developed waterborne dispersions have been assessed by dynamic light scattering (DLS), Z-potential, and accelerated aging tests (analytical centrifugation). Subsequently, selected fabrics have been face-coated by the WPUD using knife coating method and their properties have been assessed by measuring water contact angle (WCA), water column, fabric stiffness, and air permeability. The electrical conductivity of textiles coated with SWCNT-doped WPUD has been evaluated by EN 1149 standard. Finally, the surface morphologies of uncoated and coated fabrics have been studied by scanning electron microscopy (SEM). All of the synthesized polyurethane-ureas provide the coated substrates with remarkable water-repellency and water column, being therefore a more sustainable alternative to waterproof coatings based on fluoropolymers, such as PTFE. The additivation of the polymeric matrices with SWCNT has led to textile coatings with excellent electrical conductivity, maintaining water column properties, giving rise to multifunctional coatings that are highly demanded in protective workwear and technical textiles.

## 1. Introduction

Hydrophobicity, permeation-resistance, and electrical conductivity are highly valued functionalities for technical textiles, like medical fabrics, protective workwear, technical garments for extreme outdoor sports, automotive and aeronautical textiles, wearable sensors, smart textiles, etc. Perfluoroalkyl and polyfluoroalkyl substances (PFASs) have been, for several decades valuable, widely used chemicals to coat textile substrates, as they are inert, low surface energy materials, therefore providing coated fabrics with exceptional durable water and oil repellence (DWOR). PFASs can be divided in two classes: non-polymeric and polymeric PFASs [1]. The most commonly used PFASs in the textile industry fall within the last class. As defined by Buck et al. [1], polymeric PFASs encompasses fluoropolymers, like polytetrafluoroethylene (PTFE) or polyvinylidene fluoride (PVDF), side-chain fluorinated polymers (commonly known in the textile sector as fluorocarbons), and perfluoropolyethers.

It is well established that non-polymeric PFASs, like perfluorooctanoic acid (PFOA) and perfluoroctane sulfonic acid (PFOS), possess harmful effects on health and the environment [2,3,4,5,6]. Shorter homologues, like perfluorohexanoic acid (PFHxA), are currently under study to better understand their environmental and human health effects [7,8], and it is likely that restriction proposals that were recently posted by some EU countries will come into force in the near future. On the other hand, polymeric PFASs are a potential source of non-polymeric PFASs and bioavailable small particles [9,10]. Fluoropolymers, like PTFE and PVDF in the form of emulsions or fine powders, are manufactured using non-polymeric PFASs as processing aids that can be released to the environment during their entire life-cycle, which is, manufacture, application on fabrics (manufacture of finished articles), use, and disposal [11]. Furthermore, many fluoropolymers are commercialized in the form of fine suspensions of low particle size that can give rise to potentially dangerous fluorinated particles and oligomers [12,13]. In addition, side-chain fluorinated polymers can degrade and release non-polymeric PFASs to the environment [14] and, therefore, are considered to be high concern chemicals.

**Figure 1:**
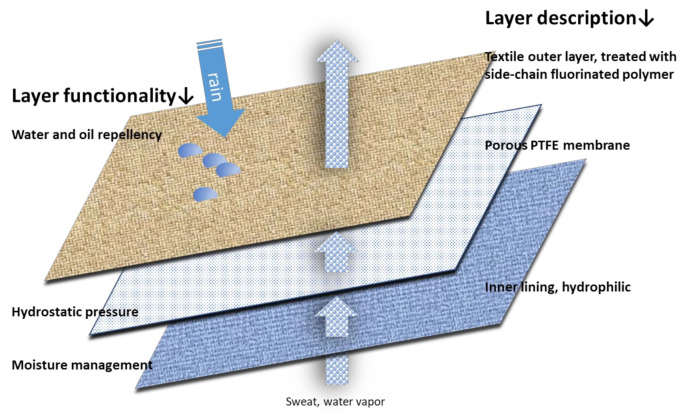
Scheme of a typical multi-layered fabric for outdoor clothing.

It should be noted that commercial waterproof textiles usually contain multiple types of PFASs susceptible to being released into the environment [15]. For instance, the multiple layered materials used in all-weather clothing are the result of a laminate commonly composed of three layers: outer layer made of polyester or polyamide fibers that are coated by a side-chain fluorinated polymer to confer water and oil repellence, middle layer, which is typically a PTFE or polyurethane membrane that confers hydrostatic pressure, and, finally, an inner layer of hydrophilic nature, which allows the transport of sweat and moisture to the outside. See Figure 1 for a schematic of a typical multi-layered fabric for outdoor clothing. All of the above-mentioned concerns regarding PFASs in textile sector are leading to intensive research and high market demand of fluorine-free alternatives for DWOR finishing [16,17,18].

On the other hand, providing fabrics with electrical conductivity without affecting the rest of the material’s properties is a highly demanded feature. To achieve this goal, polymeric fibers that are made of conductive polymers or polymeric filled nanocomposites have been studied, but this alternative is expensive and the use of high content of fillers can lead to fibers with poor mechanical properties, poor processability, and high cost. Another alternative is the use of coatings to make conductive textiles. Several technologies, such as layer-by-layer deposition [19], electroless plating [20], or chemical plating [21], among others, have been explored, but with several drawbacks, such as the pollution by heavy metals, time-consuming, and expensive processes [22]. In this context, the development of sustainable conductive coatings, which can be also a reliable alternative regarding PFASs for providing water repellence and water column to the textiles, is an appealing research for the textile industry. Bio-based, waterborne polyurethanes are eco-friendly in nature, avoiding the use of traditional solvents that are toxic and expensive. They also include bio-based building blocks in its composition, and its structure can be tailored to meet specific applications [23,24,25]. Moreover, they can be modified with different nanofillers to provide them with new properties. Carbonaceous nanostructures, such as carbon nanotubes (CNTs) or graphene, have been reported as a suitable nanofillers for providing conductivity to textile coatings, owing to their unique electrical properties. However, the property improvements can be limited due to the degree of dispersion or poor orientation of the nanomaterial [26]. Different approaches have been reported for improving their dispersion or orienting carbon nanofillers by employing mechanical stretching [27], an electric field [28], or a magnetic field [29], but these approaches are usually difficult to apply at the industrial level.

This work focuses on providing a sustainable and multifunctional alternative to fluoropolymers, like PTFE or petrol-based polyurethanes, which are commonly employed as a membrane to confer water column to multi-layered fabrics for High-Tech applications. Firstly, a series of fluorine-free, waterborne, partially bio-based polyurethane-urea dispersions (WPUD) with remarkable hydrophobicity and good resistance to water permeation (water column or hydrostatic pressure) has been synthesized, characterized, and applied to fabrics by knife coating to form a membrane on its surface. The incorporation of single-walled carbon nanotubes (SWCNT) in the polyurethane-urea matrix by the use of a commercial masterbatch has also been studied as a simple strategy to confer electrical conductivity to the coatings.

The partially bio-based polyurethane-ureas were obtained by the prepolymer method using, as starting building blocks, isophorone diisocyanate (IPDI), 100% bio-based polyester-polyol (Priplast 3294), internal emulsifier dimethylolbutanoic acid (DMBA), and chain extenders bio-1,3-propylene glycol (1,3-PDO) and ethylene diamine (EDA). The experimental design of ternary mixture has been applied as a methodology to explore systematically the ratio between polyol, internal emulsifier and chain extenders.

Structure and properties of the obtained polyurethane-ureas have been assessed by FTIR, NMR, DSC, TGA, DMTA, and stress–strain mechanical tests. Moreover, DLS, Z-potential, and analytical centrifugation have been used to assess particle size distribution and the stability of the developed waterborne dispersions. Subsequently, selected fabrics have been face-coated by the WPUD by knife coating, and their properties have been assessed by measuring water contact angle (WCA), water column, fabric stiffness, and air permeability. The electrical conductivity of the coated textiles with SWCNT-modified WPUD has been evaluated by the EN 1149 standard. Finally, the surface morphologies of uncoated and coated fabrics have been studied by scanning electron microscopy (SEM).

## 2. Materials and Methods

### 2.1. Materials

**Figure 2:**
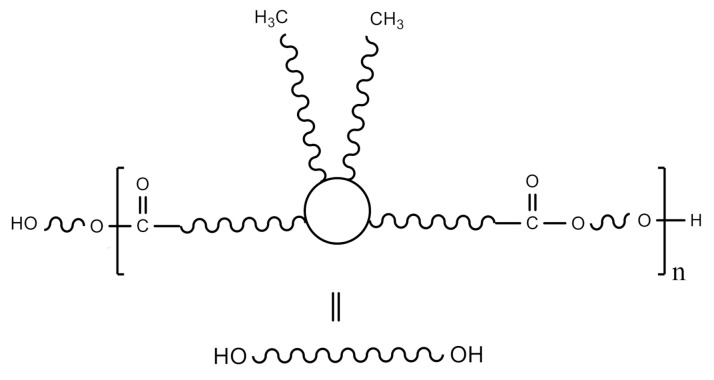
The chemical structure of Priplast 3294.

A fully biobased semicrystalline polyester polyol that was based on dimerized fatty acid (Figure 2), commercially known as Priplast 3294 was supplied by Croda Iberica. Priplast 3294 is a highly-hydrophobic, semicrystalline polyester polyol, providing high flexibility at low temperatures, good hydrolytic stability, and enhanced adhesion to dissimilar substrates. This polyol has a M_n_ of 2000 g mol^−1^, f_(OH)_ = 2, and an average hydroxyl value of 56 mg KOH g^−1^. Isophorone diisocyanate (IPDI, ≥99.5% purity) was supplied by Evonik Industries GmbH (Vestanat IPDI). 2,2-Bis(hydroxymethyl)butyric acid (DMBA, 99% purity) was supplied by Anhui Sinograce Chemical CO., LTD. 1,3-Propanediol (1,3-PDO, ≥99% purity and 100% biobased) was supplied by DuPont Tate&Lyle BioProducts. Acetone (≥99.5% purity), triethylamine (TEA, 99% purity), and ethylenediamine (EDA, ≥99% purity) were purchased from Sigma-Aldrich. Everchem Specialty Chemicals supplied Bicat 8108 (bismuth neodecanoate 20%).

The additives to formulate the printing pastes were supplied by Color Center, S.A. and are: Defoamer PR (defoamer, mineral oil-based), Complex DG (diethylene glycol-based, runnability improver), DMEA (dimethylethanolamine, neutralizing agent), and Thickener L-120 (polyacrylic acid, associative thickener). A commercial aliphatic polyether-urethane waterborne dispersion from Color Center, S.A., 35% solids, non-biobased, referenced as RD27, has been included in this work as a comparative example.

A masterbatch containing 10 wt. % SWCNT dispersed in a wax matrix, TUBALL MATRIX BETA 302, easily dispersible in waterborne systems, was employed to provide polymeric dispersions with electrical conductivity. The sample was kindly supplied by OCSiAl EUROPE.

Pristine polyester fabric (UPRON) was supplied by HEDVA a.s. and it was used as a substrate to perform the coatings. The main characteristics of the pristine UPRON fabric are summarized, as follows: 100% polyester fabric (PET, polyethylene terephthalate), plain weave, 172 ± 5 g m^−2^; and, threads per cm^2^: warp 32 ± 2, weft 14 ± 1.

### 2.2. Synthesis of Waterborne Polyurethane-Urea Dispersions

Priplast 3294 was dried under vacuum at 110 °C and 35 mbar for 1 h before use. Priplast 3294 was introduced into a 750 mL five-necked reactor fitted with a mechanical stirrer, an inlet for nitrogen, and a reflux condenser. DMBA internal emulsifier and PDO chain extender were added to the reactor and degassed for 1 h at 80 °C under stirring to complete homogenization. IPDI and Bicat 8108 (25 mg per kg of mixture) were subsequently added to the reactor. The temperature was maintained at 80 °C under nitrogen blanketing until the theoretical value of NCO was reached (measured by titration using the dibuthylamine method). The reaction time for the prepolymer formation was between 2 and 2.5 h. All of the prepolymers were prepared at an isocyanate/hydroxyl ratio (NCO/OH) of 1.62. Afterwards, acetone was added to decrease the viscosity of the prepolymer and facilitate its subsequent dispersion in water (typically 100 g/200 g of prepolymer). The reaction mixture was cooled to 40 °C and TEA was added slowly though a dropping funnel and the mixture maintained for 30 min. under stirring to ensure complete neutralization of the carboxyl groups from DMBA. The reaction system was subsequently cooled to 30 °C and cold deionized water at 8 °C was quickly added with vigorous stirring to promote phase inversion, thus obtaining a milky dispersion of the prepolymer in water. Chain extension agent EDA was stoichiometrically added to react with the free isocyanate groups of the prepolymer, previously diluted in water to 20%, drop-by-drop by keeping the temperature of the mixture between 15 and 18 °C, gentle stirring for an additional 1 h. Finally, the biobased WPUD was obtained after removing acetone by a rotary evaporator under reduced pressure (250 mbar) at 60 °C. All of the synthesized WPUD were adjusted to a solid content of 35%. It is worth noting that acetone can be readily recovered and recycled.

**Figure 3:**
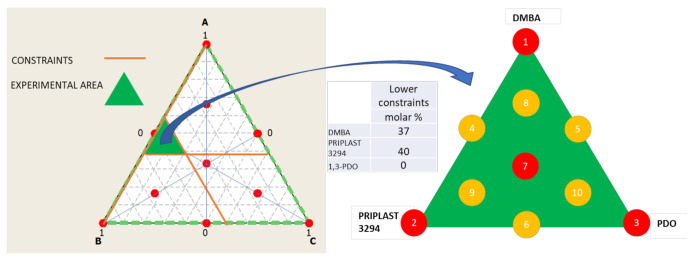
Ternary mixture design of experiments employed to study the proportion between the tree diols that form part of the polymer. (**Left**): in white full design surface area with all the theoretical experimental points marked in red and studied area in green which is delimited by the lower constraints stablished for component A (DMBA) and B (Priplast). Middle: table summarizing the lower constraints stablished for the diols. (**Right**): area studied and experimental points of de simplex design plot (from 1 to 7), experiments marked in red have been performed in this work (1, 2, 3, 7) while experiments that are marked in yellow have not been carried out.

The ratio between the three diols used in the prepolymer synthesis (Priplast 3294, DMBA, and 1,3-PDO) was systematically varied using the methodology of ternary mixture design of experiments (Figure 3). In our particular case, lower constraints were applied to DMBA and Priplast 3294 because, due to the particularity of the system, it is not possible to study the proportions of the three diols in the full range (from 0 to 100 molar %). For example, the amount of DMBA could not be equal to 0, since then the final polymer would not be dispersible in water; therefore, the lower constraint for DMBA was stablished in 37 molar %. A minimum amount of Priplast was also established, so that all of the prepolymers contain at least a 40 molar % of this polyol.

Table 1 shows the overall simplex design table, with all of the experiments that could be done to fully cover the study area of ternary mixture (simplex ternary mixture design). The five columns on the right show the molar % of each polyol employed in the synthesis considering the lower constraints, hard segment content in wt. %, and bio-based content of each experiment in wt. %. The experiments that have been performed in this work have been named starting with 3294IPDI-, and the rest of experiments have not been carried out.

Polyurethane-urea films, suitable for NMR, mechanical, thermal, and swelling characterization were prepared by carefully pouring 20 g of WPUD in a circular Teflon mold, letting the water to evaporate slowly at 40 °C into a vented oven for 48 h to obtain 1 mm thick films that were subsequently cured and dried at 60 °C for 48 h in a vacuum oven.

### 2.3. Additivation of WPUD with SWCNT

The preparation of a WPUD with 0.1% content in SWCNT: 2 g of Tuball Matrix Beta 302 was poured in a cylindrical mixing beaker with a flat bottom and a diameter of 10 cm, 2 g of the selected WPUD were added and then manually dispersed with the help of a glass rod, gently stirred for 15 min. until complete homogenization. Afterwards, 98 g of the same WPUD were added slowly and the mixture was subjected to mechanical stirring with an impeller blade (diameter of 5 cm) and a peripheral speed of 10 m s^−1^ (Heidolph RZR series) for 20 min. Finally, another 100 g of the WPUD were added and the same stirring was continued for 20 min.

To obtain a WPUD with a SWCNT content of 0.05 wt. %, 100 g of the WPUD with 0.1% content in SWCNT were thoroughly mixed with 100 g of the non-additivated WPUD. The mixing procedure was performed using the same beaker and mechanical stirrer discussed above, stirring for 20 min. to ensure complete homogenization.

### 2.4. Application of WPUD on Fabrics by Coating

The fabrics were coated by the printing pastes that were made of WPUD, water, defoamer, runnability improver, DMEA, and thickener (see Table 2 for the standard printing paste formulation, solid content, and viscosity).

Knife coating procedure, which is a widely used coating method in the textile sector, was employed to coat all of the fabrics with the corresponding printing pastes. Knife coating was performed using a laboratory coating machine R2R continuous line Werner-Mathis in a coating regime, air knife 90°, followed by drying in vented-oven at 110 °C at a speed of 1 m min.^−1^ and curing at 150 °C at a speed of 0.4 m min.^−1^.

The dry add-on of the coated fabrics was calculated, as follows: a sample cutter James H. Heal model 230/100 was used to cut out regular circular specimens of fixed area (100 cm^2^) from the uncoated fabric and all of the coated fabrics. The calculation of weight per square meter (grammage, G) of a given specimen is performed by multiplying the specimen’s weight measured by a balance with a readability of 0.01 g by a factor of 100. Finally, the dry add-on of a coated fabric is calculated using Equation (1), where Gc and Gu are the grammages of the coated and uncoated specimens, respectively.

Dry add on = Gc − Gu (1)

### 2.5. Characterization Techniques

#### 2.5.1. Characterization of Synthesized Polymers and Dispersions

Fourier transform infrared spectroscopy (FTIR) was employed for chemical characterization of the WPUD. The FTIR spectra were obtained using a Perkin Elmer Spectrum Two equipped with transmission accessory. A drop of each waterborne dispersion was spread on a SeZn FTIR window and then dried under IR lamp to evaporate water and to obtain a thin film. Eight scans were taken for each sample in the range of 4000–500 cm^−1^ with a resolution of 4 cm^−1^.

The structural characterization of all the WPUD casted films was performed by ^1^H-NMR, using a Bruker AMX-300 spectrometer at 25 °C operating at 300.1 MHz. Samples were dissolved in deuterated chloroform or deuterated tetrachloroethane, and spectra were internally referenced to tetramethylsilane (TMS). Approximately 10 mg of sample dissolved in 1 mL of solvent was used to collect the ^1^H-NMR spectra. Sixty-four scans were acquired with 32 K data points as well as relaxation delay of 1 s.

Differential scanning calorimetry (DSC) studies of the dry films of all the synthesized WPUD as well as Priplast 3294 have been carried out to determine their melting temperature (*T_m_*), crystallization temperature (*T*_c_), melting enthalpy (Δ*H*_m_), and crystallization enthalpy (Δ*H*_c_), as well as possible second-order transitions such as glass transition temperature (*T*_g_). The samples were heated from −90 to 150 °C at a constant heating rate of 10 °C/min in a Perkin Elmer model Pyris I under nitrogen atmosphere (50 mL/min.), working with 5 mg samples placed in sealed aluminium pans. The *T*_g_ values were determined by the StepScan DSC technique that yields enhanced characterization information by separating out the reversible and irreversible thermal events. The measurements were run from −100 °C to 100 °C at heating and cooling rates of 4 and 2 °C/min., respectively, a modulation of amplitude of 1 °C and period of 60 s.

The thermal stability of the synthesized WPUD films has been studied by thermogravimetric analysis (TGA), using a Mettler Toledo TGA2 equipment. The thermogravimetric analysis consisted of recording the weight loss of the samples that were subjected to a temperature gradient from 25 °C to 600 °C at 10 °C min.^−1^ in a furnace with nitrogen atmosphere.

Dynamic Mechanical Thermal Analysis (DMTA) was carried out on rectangular samples 10 × 11.06 × 0.1 mm cut from all of the WPUD films to determine the storage modulus (G’) and the loss factor tan δ. The specimens were strained at 0.05% at 1 Hz frequency, using single cantilever clamp TA Instruments DMA Q800 working under tension mode. They were scanned at a heating rate of 3 °C min.^−1^ from 30 °C to 160 °C, previous equilibration at 25 °C for 5 min.

Mechanical properties were determined by stress–strain tensile measurements. The tests were carried out following the BS ISO 37: 2005 standard, using a Zwick/Roell model 500 N equipment. The measurements were carried out on dumbbell shaped Type 4 specimens cut from the WPUD films. Dumbbell dimensions are summarized in Figure S9 of Supporting Information. The test conditions were, as follows: preload 0.1 MPa, preload speed 1 mm min^−1^, and test speed 50 mm min.^−1^. For each WPUD, at least five samples were taken in different parts of the films and tested. The characteristic parameters measured were: elastic modulus (E), stress at break (σ_b_), deformation at break (ε_b_), and stress at 100% strain (σ_100%_).

Water-swelling measurements were performed using dumbbell shaped Type 4 specimens cut from the WPUD films. The samples were placed in a closed vial with 20 mL of deionized water at 25 °C for 48 h. Swelling degree was determined by Equation (2), where *w*_0_ and *w* were respectively the weight of the initial dried material and this of the swollen material after 48 h. The experiments were carried out in triplicates for each specimen.

(2) $$Swelling\;\left(\%\right)=\frac{w-w_{0}}{w_{0}}\times 100$$

The pH of each WPUD was measured with a HACH sensION+ MM378 pH meter that was calibrated with pH = 4 and seven standard solutions.

Moreover, the particle size and particle size distribution (PSD) of developed aqueous polymeric dispersions have been characterized by Dynamic Light Scattering (DLS). The stability of the dispersions has been analyzed by Z-potential measurements. DLS and Z-potential tests were performed on a Malvern Zetasizer ZS equipment at 20 °C. Z-potential measurements were performed after the dilution of the WPUD to 5 wt. % with deionized water buffered at pH 8.2, while the particle size measurements were performed after dilution of the WPUD to 10 wt. % with deionized water.

The WPUD were also subjected to accelerated sedimentation tests using an analytical centrifuge LUMiFuge 110–153.3–12 (LUM GmbH, Berlin, Germany) in order to evaluate long term stability. In each measurement, the suspension was pipetted into a polyamide transparent cell with a path length of 2 mm and 10 mm. Initially, the separation velocity of the WPUD emulsions with the relative centrifugal forces has been analyzed up to 2000× *g* (500, 1000, and 2000× *g*), with changes in the sedimentation boundary not being not observed. Thereafter, measurements were performed at 4 and 40 °C and relative centrifugal force of 2000× *g*, with a scanning rate of one every 10 s for 5 h. When considering a linear dependency of the WPUD dispersions, the duration of the tests have been optimized to simulate a shelf-life of 14 months, in accordance with ISO/TR13097 [30]. Moreover, a representative sample has been analyzed one year after the synthesis to determine long-term stability.

#### 2.5.2. Characterization of Coated Textiles

The water repellence of the coated fabrics has been tested by measuring the contact angle of a droplet of water placed on the surface of the coated textile. Water contact angle measurements (WCA) were carried out under ambient conditions with a SURFTENS Universal automatic goniometer. Static contact angle measurements and advancing and receding contact angles of the air–water interface were measured on each sample. For each test, an average value of five measurements was adopted as the value of WCA. For the water static contact angle measurement, a drop of deionized water with a volume of 5 μL was placed on the textiles. For the advancing (θ_Av_) and receding angle (θ_Re_) measurements, first, a 5 µL of de-ionized (DI) water droplet was placed on the surface using a needle. Next, another 5 µL DI water was pumped into the initial droplet at a constant rate for the advancing contact angle measurement. A contact angle measurement was obtained after inflating the drop with every 0.1 µL. The advancing contact angle value was given as the average of the obtained 51 values. Subsequently, 5 µL DI water at the same speed was removed from the existing droplet to measure the receding contact angle. The receding contact angle was obtained as the average of the 51 values obtained after removing every 0.1 µL from the droplet.

The characterization tests summarized in Table 3 and Table 4 were performed according to the indicated standards.

The surface morphologies of the relevant coated and uncoated fabrics were studied by scanning electron microscopy (SEM) using an Ultra Gemini-II microscope from Carl Zeiss SMT.

## 3. Results and Discussion

### 3.1. Synthesis and Characterization of WPUD

**Figure 4:**
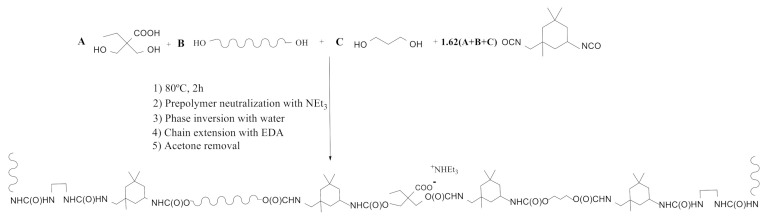
Synthesis scheme of the waterborne polyurethane-urea dispersions.

A series of WPUD were synthesized by the prepolymer method, as reported in the experimental section. Once the prepolymer was obtained, the neutralization of the carboxyl groups with triethylamine and subsequent phase inversion in water was carried out. Finally, chain extension by EDA was performed, followed by acetone removal by rotary evaporation and standardization to 35% solid content. The scheme of the synthesis strategy of the WPUD can be seen in Figure 4.

**Figure 5:**
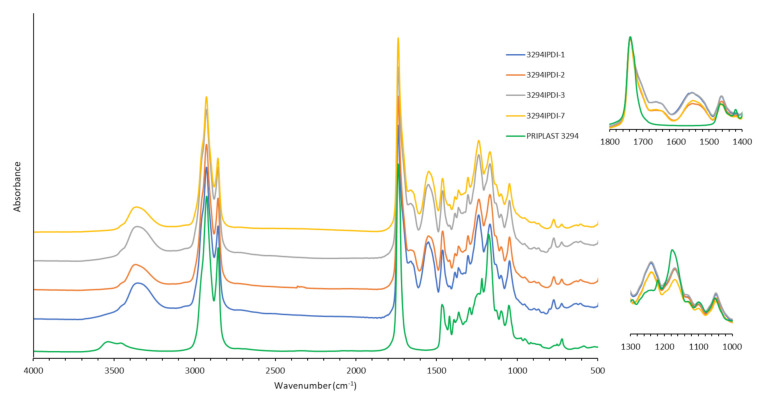
FTIR spectra in the MIR region for synthesized WPUD films. Magnifications of the 1800–1400 cm^−1^ and 1300–1000 cm^−1^ regions.

WPUD were first characterized by FTIR. The infrared spectra of all synthesized polymers showed the complete conversion of isocyanate groups judging by the absence of the characteristic free isocyanate band at 2275 cm^−1^. By comparing the spectrum of the starting polyol Priplast 3294 with those of the synthesized polyurethane-ureas, the appearance of new bands that are indicative of the formation of urethane/urea bonds can be clearly confirmed (Figure 5). The broad band of NH asymmetrical and symmetrical stretching vibration at 3351 cm^−1^ indicates the great extent of NH established hydrogen bonds with carbonyl groups from urethane, urea, ester, and ionic carboxylate from internal emulsifier DMBA [31]. The zoom in the area of 1500–1600 cm^−1^ shows NHCO stretching and NH bending bands of urethane group at 1550 cm^−1^, as the hard segment content in the polymer increases, the intensity of this bands also increases; this observation is in good agreement with Poussard et al. [24]. Further enlargement in the area between 1740 and 1600 cm^−1^ shows characteristic C=O stretching bands from DMBA carboxylate, urethane, and urea carbonyl groups at approximately 1661–1700 cm^−1^ region, partially overlapped with the ester band from the polyol at 1739 cm^−1^. COC(O) stretching bands and NH out-of-plane bending bands from urethane functional groups at 1242 cm^−1^ and 774 cm^−1^, respectively, are clearly observed.

**Figure 6:**
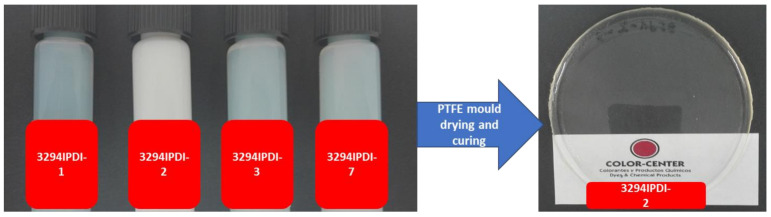
Appearance of the synthesized WPUD (**left**) and appearance of casted film from one of the polyurethane-urea dispersions (3294IPDI-2, **right**).

All of the synthesized WPUD were dried on Teflon plates to obtain the corresponding films. All of the obtained films were transparent and homogenous and were used for NMR, mechanical, thermal, and swelling characterization. Figure 6 shows the appearance of the synthesized WPUD as well as the appearance of casted film from one of the polyurethane-urea dispersions (3294IPDI-2).

**Figure 7:**
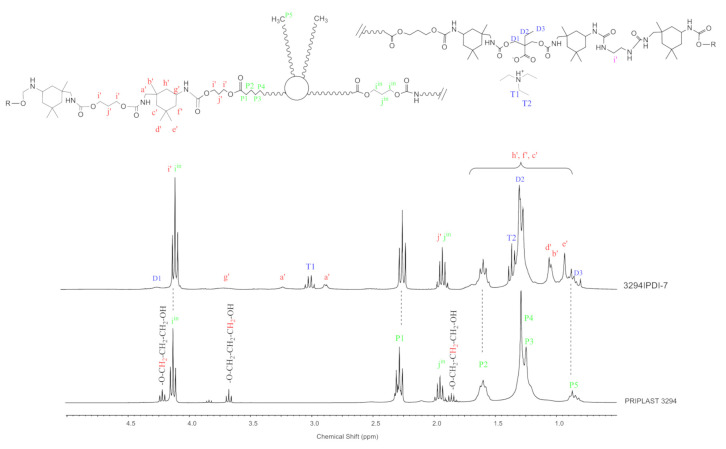
^1^H NMR of experiment 3294IPDI-7 and starting polyol Priplast 3294 with peak assignments.

^1^H NMR spectra of all polymer films were recorded, thus confirming chemical structure. ^1^H NMR spectra of all WPUD films are reported in Figure S1 of Supporting Information. Figure 7 depicts the ^1^H NMR of experiment 3294IPDI-7 and starting Priplast 3294 polyol with peak assignments.

^1^H NMR spectra provided structural information regarding Priplast 3294 polyol and WPUD films. The main peaks of Priplast 3294 are in good agreement with those reported by Bueno-Ferrer’s work [32], showing CH_2_C(O)O signal at 2.30 ppm and peaks from CH_2_OC(O) and CH_2_ in the β position of the ester group at 4.15 and 1.61, respectively.

The CH_2_ peaks from the methylene in the α, β, and γ position with respect to free hydroxyl groups (COO-C_γ_H_2_-C_β_H_2_-C_α_H_2_-OH) appear at 3.69, 1.96, and 4.23 ppm, respectively. In the WPUD, the full conversion of hydroxyl groups to form polyurethane segments is confirmed by downfield shifting of the α and β peaks in the polymer spectra.

Commercially available IPDI represents an isomer mixture of approximately 75:25 in favor of the cis- isomer, leading to different reaction mixtures [33] and resulting in complex ^1^H-NMR spectra. For a better interpretation of the ^1^H-NMR spectra of the synthesized polyurethane-ureas, they were compared with the ones of model compounds (IPDI:DMBA, IPDI:PDO, IPDI:Priplast 3294, IPDI:EDA, IPDI:EtOH) that were obtained by reacting IPDI with the corresponding building block in a molar ratio of 2:1. Supporting Information (Figures S2–S6) shows the interpretation of the ^1^H-NMR spectra of these model compounds. This has made it possible to more precisely assign some of the main signals of the polyurethane-ureas under study.

Urethane moieties give the following weak signals:

- IPDI CH_2_ in α-position to -NHC(O) group (a’), 3.24 ppm and 2.88 ppm corresponding to trans isomer (25% abundance) and cis isomer (75% abundance), respectively.
- IPDI CH in α-position to -NHC(O) group (g’), 3.73 ppm.

The incorporation of internal emulsifier (DMBA) into the polymer backbone can be assessed by the following signals: DMBA CH_2_ in α-position to OC(O) group (D1), 4.26 ppm; DMBA methylene group (D2) attached to methyl, 1.31 ppm; DMBA methyl group (D3), 0.86 ppm.

Triethylammonium salt can be assessed by the peaks at 3.02 (T1) and 1.37 (T2) ppm that correspond to CH_2_ and CH_3_, respectively, of ethyl group.

**Figure 8:**
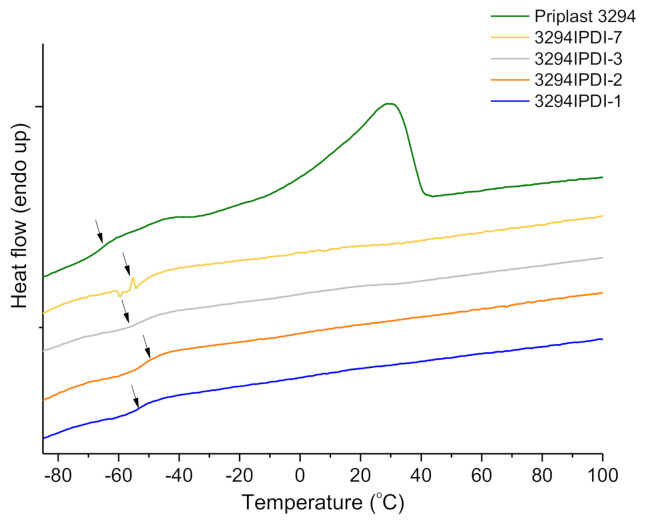
DDSC thermograms of Priplast 3294 and WPUD films. Glass-transition temperatures has been calculated taken as the inflection point of the heating step scan DSC.

The thermal properties of the developed WPUD have been analyzed by DSC and TGA. The non-isothermal DSC thermograms for all of the WPUD films as well as Priplast 3294 have been registered. The DSC thermogram of Priplast 3294 displayed a clear melting peak at 33.0 °C, and a crystallization peak at −6.2 °C, with fusion and crystallization enthalpies of 41.0 and 38.3 J/g, respectively, thus confirming its semicrystalline nature attributed to lamellar packing of methylenes of both paraffinic backbone and side chains (see DSC of Priplast 3294 in Figure S7 of Supporting Information). For the WPUD films, only glass transition temperatures have been detected and determined by DDSC, see Figure 8 and Table 5. The glass transition temperatures (*T*_g_) of the soft segments are very close to the value observed for Priplast 3294 polyester polyol. This is an indication that there is a phase segregation between hard polyurethane-urea segments and polyester soft segments. This phase segregation is enhanced as the content in hard segments increases, and it is reflected by a small decrease in the *T*_g_ values observed in the studied series.

**Figure 9:**
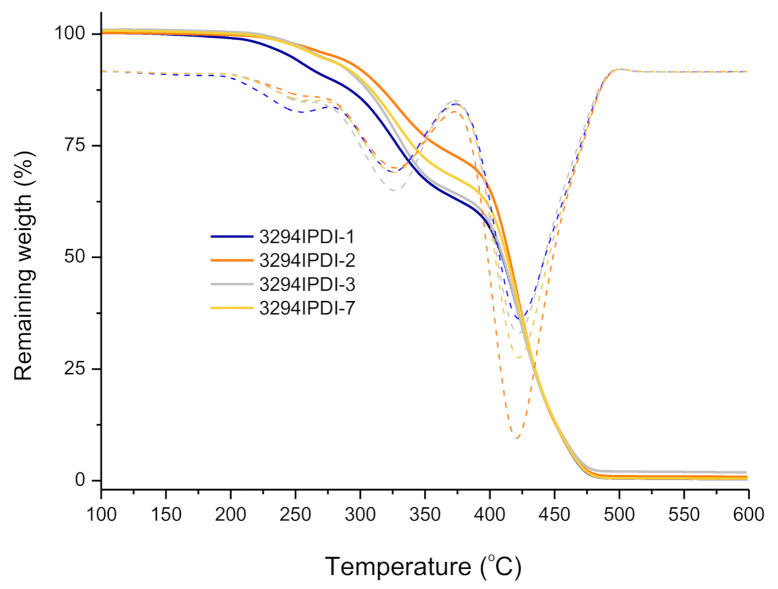
TGA thermograms of synthesized WPUD films. Mass losses (thick lines) and derivative curves (dashed lines).

The thermal stability of the synthesized WPUD films has been studied by TGA and the curves are collected in Figure 9. The degradation temperatures corresponding to a weight loss of 10% and the temperatures of the maximum degradation rate for each degradation stage are collected in Table 5. All of the (co)polymers have sufficient thermal stability to withstand without degradation the temperature conditions that are required during coating procedures.

As can be observed in Figure 9, thermal degradation occurs in three stages, with similar temperatures of maximum degradation rate for all the synthesized WPUD. First step occurs in the temperature range 255–264 °C and could be attributed to volatilization of triethylamine which is in the form of carboxylate salt. Stage 2 around 326 °C corresponds to the degradation of urethane and urea bonds [24]. The remaining weight of WPUD film at this stage is higher for the samples with higher Priplast 3298 content, as can be observed in Figure 9. Finally, stage 3 around 422 °C is related to the degradation of segments of Priplast 3294 biobased polyol (see Figure S8 of Supporting Information). The TGA curve of commercial non-biobased polyether polyurethane film (RD27) has been performed and compared to WPUD 3294IPDI-7, see Figure S8 of Supporting Information. It can be observed that thermal degradation for RD27 also occurs in three stages, and the temperature of maximum degradation rate for the third stage is around 400 °C, thus being 20 °C lower than that observed for 3294IPDI-7. These differences in thermal degradation can be attributed to the different chemical structure of the polyol (polyether vs. polyester).

**Figure 10:**
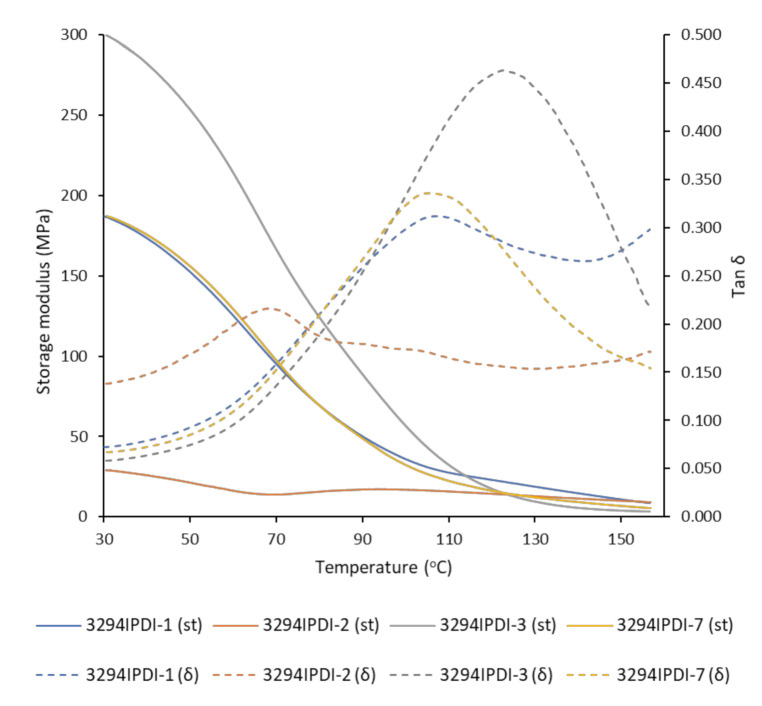
DMTA curves of synthesized WPUD films. Storage modulus (full lines) and tan δ (dashed lines).

**Figure 11:**
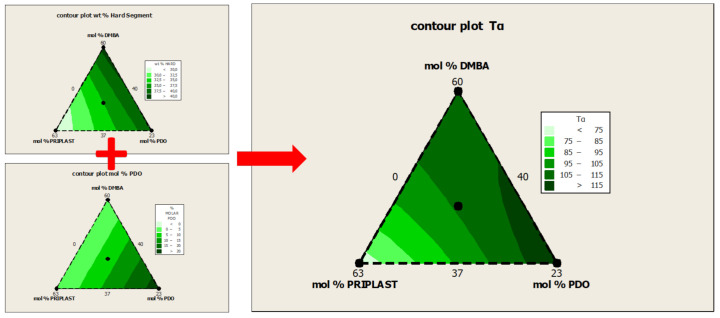
Contour plots obtained by Minitab software from the ternary mixture experimental design. (**Left**) (**up**): contour plot of Hard segment content in wt. %. (**Left**) (**down**): contour plot of 1,3-PDO content in mol %. (**Right**): contour plot of relaxation temperature (*T*_α_).

The dynamomechanical behavior was studied for all of the obtained WPUD films. Figure 10 shows the plots of G’ modulus and tan δ vs. temperature in the range from 30 to 160 °C. The relaxation temperatures (*T*_α_), calculated from the maximum value of tan δ peak, are reported in Table 5. There is a good agreement between *T*_α_ and hard segment content. The highest the hard segment content the highest the *T*_α_ value due to the increase in mobility restriction that is induced by urethane and urea groups present in the rigid phase. Thus, *T*_α_ for 3294IPDI-2 (28 wt. % hard segment, HS) is the lowest within the experiment set (69.9 °C). while *T*_α_ for 3294IPDI-1 and 3294IPDI-3 (41 wt. % HS and 39 wt. % HS, respectively) are the highest within the experiment set (109.2 and 123.5 °C, respectively). From the contour plot of *T*_α_, as in Figure 11, it can be observed that 1,3-PDO also has an important influence in *T*_α_; therefore HS and 1,3-PDO both contribute to the increase of *T*_α_ values. It can be inferred from these results that hard segments that are composed of urethane groups generated from 1,3-PDO could interact more strongly by hydrogen bonding than the ones composed of DMBA, most probably due to the steric hindrance caused by the ethyl and carboxylate side groups that are present in the latter.

**Figure 12:**
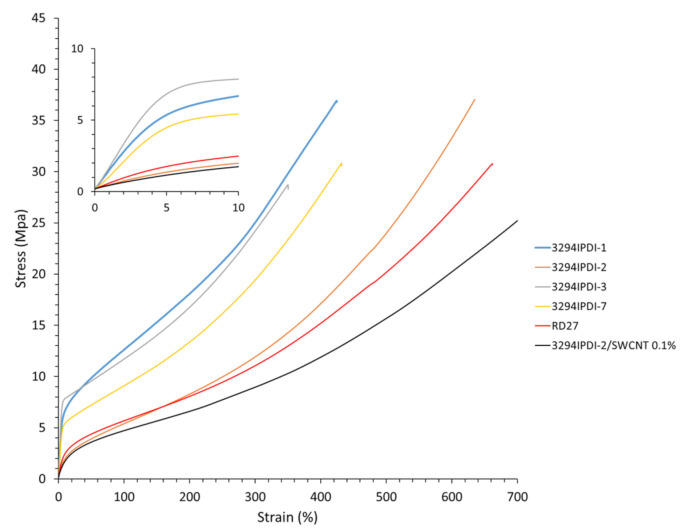
Stress–strain curves of WPUD films. Commercial non-biobased polyurethane (RD27) and polyurethane matrix 3294IPDI-2 additivated with 0.1 wt. % SWCNT (3294IPDI-2/SWCNT 0.1%) has been included for comparison.

Stress–strain tensile experiments were carried out to evaluate the mechanical properties of the synthesized WPUD films. The RD27 film has also been evaluated for comparison purposes. Figure 12 shows the stress–strain curves and Table 6 collects the main mechanical properties of films.

**Figure 13:**
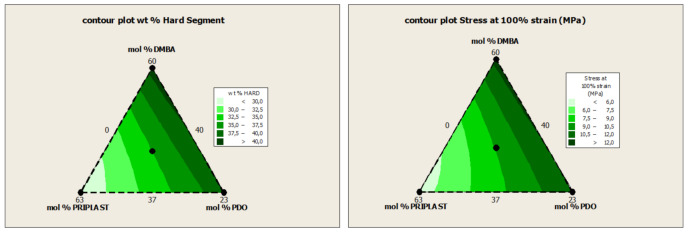
Contour plots obtained by Minitab software from the ternary mixture experimental design. (**Left**): contour plot of Hard segment content in wt. %. (**Right**): contour plot of σ_1__00%_ (stress at 100% strain).

**Figure 14:**
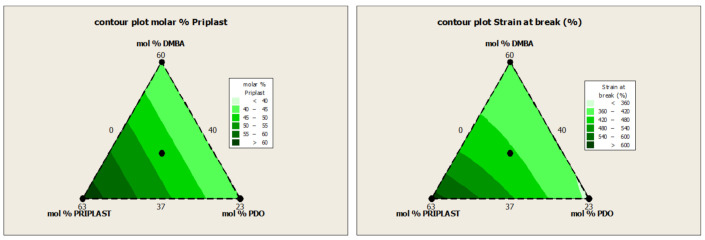
Contour plots obtained by Minitab software from the ternary mixture experimental design. (**Left**): contour plot of Priplast 3294 content in molar %. (**Right**): contour plot of ε_b_ (strain at break).

The ratio of Priplast 3294, 1,3-PDO and DMBA in the polyol ternary mixture determined polyol composition in each synthesized polymer and, therefore, the hard segment content. There is a reasonably good match between the Young modulus and hard segment content. In general, higher hard segment content showed a higher Young modulus. However, experiment 3294IPDI-3 (39 wt. % HS) led to a slightly higher Young modulus than experiment 3294IPDI-1 (41 wt. % HS), which could be explained by the greater influence of 1,3-PDO on the polymer rigidity caused by stronger hydrogen bonding interactions between urethane groups as compared to DMBA. The concordance between stress at 100% strain (σ_100%_) and hard segment content is even better, as can be seen in the surface plots Figure 13. The strain at break is also very well correlated to the amount of Priplast 3294 (the polyol that provides flexibility), the higher the ratio of Priplast with respect to 1,3-PDO and DMBA, the highest the strain at break, see the contour plots in Figure 14.

3294IPDI-2 doped with 0.1% SWCNT leads to lower Young modulus and higher strain at break than the non-doped polymer 3294IPDI-2. This variation in mechanical properties could be caused by the waxes in which the nanotubes are dispersed in TUBALL MATRIX BETA 302, rather than the nanotubes themselves, as far as those waxes would act as plasticizers [34]. Commercial non-biobased polyurethane RD27 has a similar stress–strain profile than 3294IPDI-2 (Figure 12). The use of the ternary mixture experimental design methodology for the production of polyurethanes allows us to explore, in a relatively simple way, different proportions of the building-blocks to obtain WPUD with the desired mechanical properties.

**Figure 15:**
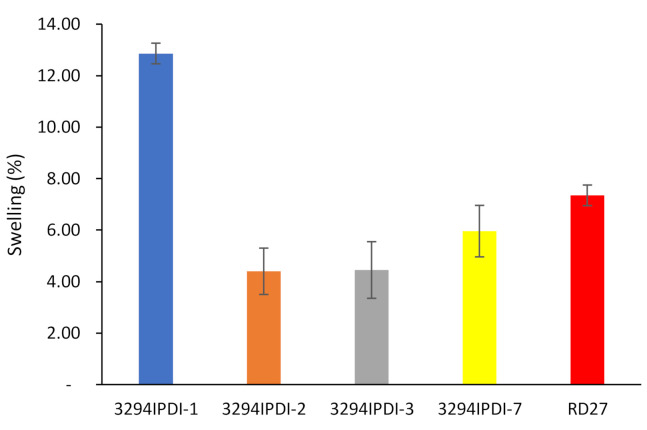
Swelling of WPUD films in water at 25 °C for 48 h.

Water swelling of the films after 48 h at 25 °C was assessed on all of the WPUD films, as well as the reference sample RD27 for comparison purposes. Figure 15 shows the water swelling values.

**Figure 16:**
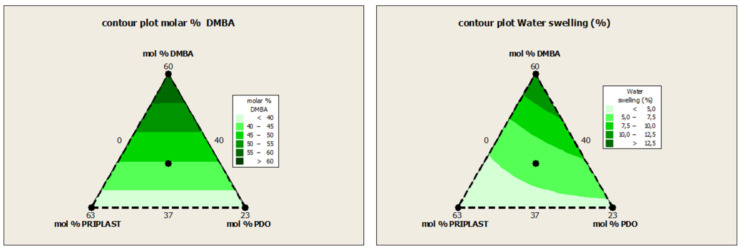
Contour plot of molar % of DMBA (**left**) and Water swelling (**right**) of all the WPUD films from de ternary mixture experimental design.

The swelling values of the films after 48 h are consistent with the increased content of hydrophilic chain extender (DMBA), in agreement with the behavior that was reported by Xu et al. for dimethylol propionic acid (DMPA) [35]. Although the DMBA content seems to be the determining factor in the water absorption of the films, the increasing content of Priplast 3294 (hydrophobic polyol soft segment) also has a notable influence in decreasing water swelling, as can be noted in contour plot from Figure 16 (right).

Finally, the dispersion stability, the particle size, and particle size distribution of WPUD have been investigated. Figure S10 of Supporting Information shows the particle size distribution (PSD) curves of all developed aqueous polymeric dispersions analyzed by DLS. Particle size distribution curves are unimodal for all WPUD. Table 7 summarizes the pH of the WPUD, average particle size, PdI values, and Z-potential. The average particle sizes were around 50 nm for all of the dispersions, except for 3294IPDI-2, which was noticeably higher and around 215 nm. This is consistent with the fact that 3294IPDI-2 has the lower content of DMBA (internal emulsifier) and the highest content on Priplast 3294 (hydrophobic polyol). The polydispersity indexes (PdI) are below 0.2, which indicated that products had satisfactory stability and good dispersibility. The smaller the value of PdI, the better the homogeneity of the dispersion [36].

The stability of the dispersions was assessed by measuring the Z potential of each WPUD. The Z potential values are collected in Table 7. For all of the 3294IPDI series, the Z potential presented similar values around -40 mV, indicating that the nanodroplets are negatively charged at the surface due to the presence of carboxylate groups. In addition, Z potential absolute values that are higher than 30 mV are generally considered to represent stable emulsions [37,38].

Accelerated sedimentation tests of WPUD emulsions were also carried out. The analysis of the tested emulsions performed at 40 °C and 4 °C and relative centrifugal force of 2000× *g* are shown in Figures S11 and S12 of Supporting Information, respectively. The first scanning profile obtained is marked in red at the bottom, and the last in green at the top. Only a small clarification is observed at the meniscus area and small sedimentations are observed at the bottom with time, whereas the light transmission of the samples remains constant with time, indicating a good stability of the emulsions. The greater the change in light transmittance during the acceleration of the emulsion, the worse the stability [39].

**Figure 17:**
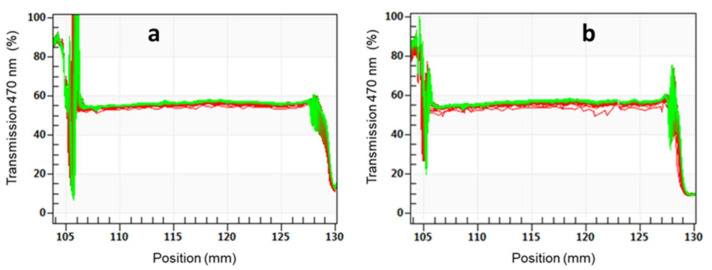
Transmission profiles of 3294IPDI-3 sample subjected to 2000× *g* and 40 °C centrifuge tests using a path length of 2 mm. (**a**) Fresh sample and (**b**) sample after one year of storage.

**Figure 18:**
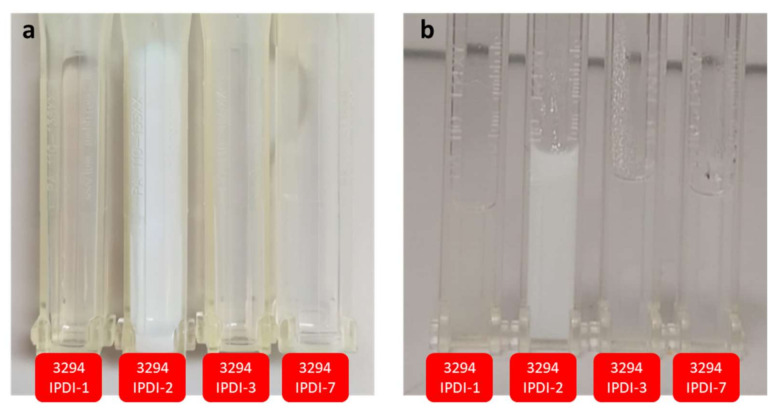
Photographs after centrifugation at 2000× *g* and 40 °C for: (**a**) fresh samples and (**b**) samples after one year of storage.

After one year of storage, WPUD emulsions were evaluated under similar conditions. Figure 17 collects the transmission profile at 470 nm of 3294IPDI-3 sample analyzed with a path length of 2 mm. Similar transmissions are observed in fresh samples and samples measured after one year of storage at room temperature for all the WPUD. The photographs in Figure 18 show the appearance of WPUD for fresh samples and samples after one year of storage, both after being centrifugated to 2000× *g* and 40 °C. No physical changes are observed in the photographs indicating the high long-term stability of the emulsions. The white aspect of 3294IDPI-2 is due to the higher average particle size in the emulsions, being previously observed in DLS measurements.

### 3.2. Additivation of WPUD with SWCNT

**Figure 19:**
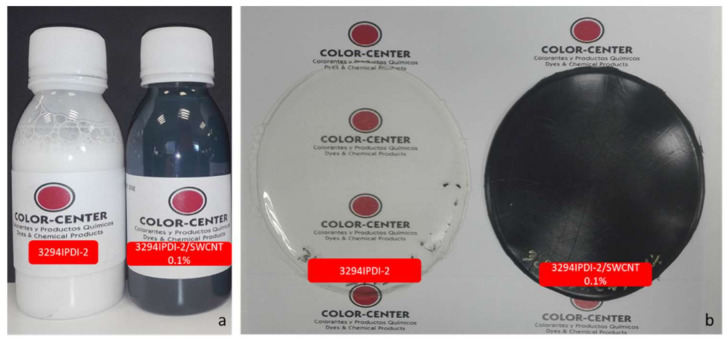
(**a**): 3294IPDI-2 WPUD dispersion before and after Tuball additivation. (**b**): casted films obtained after drying and curing in Teflon mold.

Experiment 3234IPDI-2 was selected as a polymeric matrix to disperse the SWCNT masterbatch. Figure 19 shows the appearance of 3234IPDI-2 before and after Tuball additivation as well as the casted films obtained after drying and curing in Teflon mold.

It is important to note how the additivation with SWCNT changes the appearance of the dispersion and the film that become totally black.

### 3.3. Characterization of Textiles Coated with WPUD

In order to validate the applicability and properties of the polymeric dispersions, all of the synthesized WPUD were formulated in the form of printing pastes and subsequently face-coated on 100% polyester fabrics referenced as UPRON. Knife coating was the method chosen to carry out the coatings, as it is a widely employed method in the textile sector to produce accurate and repeatable coatings, being easily scalable to standard industrial textile machinery. In this way, this work has the purpose of performing the validation of the WPUD in application conditions that are very similar to those that are commonly used at an industrial level in the sector.

Commercial non-bio-based waterborne polyurethane dispersion, referenced as RD27, and dispersions of 3294IPDI-2 doped with 0.05 wt. % and 0.1 wt. % SWCNT have been formulated as printing pastes and face-coated using same conditions than WPUD as a comparative example.

Smooth glass slides have been also coated with all of the above-mentioned printing pastes and WCA measured to establish a comparison between the glass smooth coating substrate and the inherently rough textile substrate (UPRON). The coating procedure on glass slides has been done by a more convenient manual Quadrangular Applicator with a gap of 60 µm, followed by drying at 90 °C for 5 min. and curing at 120 °C for 2 min.

Table 8 lists the values of WCA, advanced angles (θ_Av_), receding angles (θ_Re_), and calculated contact angle hysteresis (CAH) of glass slides and UPRON fabrics coated with WPUD, RD27, and SWCNT-doped 3294IPDI-2 printing pastes.

As can be observed, WCAs of coated UPRON fabrics are between 113 and 121° for the synthesized WPUD, which means low water wettability, but not superhydrophobicity (>150°). The incorporation of SWCNT in the emulsions does not affect significantly to the WCA measurements when considering the experimental deviation. Comparative example RD27 gives slightly lower WCAs and, therefore, less performance in terms of water repellence than textiles coated with WPUD and SWCNT-dopped WPUD. It is important to point out that WCAs of coated glass slides are much lower than the WCA of WPUD coated fabrics. Thus, the inherent roughness of the textile substrate contributes to achieving high values of WCA [18]. On the other hand, CAH is an estimator of the degree of imperfection of the surface as the degree of roughness and chemical heterogeneity [40,41], and it can also provide information on solid-liquid adhesion and cohesion forces. According to the low CAH values that are reported in Table 8, it could be inferred that the attractive forces between the coated fabrics (solid surface) and the water drops are low and that it is a consequence of the chemical nature of the coating.

The determination of resistance to water penetration measured by hydrostatic pressure test (also known as water column), stiffness and air permeability have been performed on UPRON fabrics face-coated with the printing pastes (Table 9). Printing paste made from experiment 3294IPDI-2 shows the highest water column value of all the 3294IPDI series, which is consistent with the mechanical properties of this experiment, having the highest ε_b_ of all the 3294IPDI series in addition to the second highest σ_b_ of all the 3294IPDI series. Therefore, experiment 3294IPDI-2 was chosen to be dopped with the SWCNT and electrostatic properties of UPRON fabrics that were coated with doped and undoped 3294IPDI-2 were measured (Table 10).

**Figure 20:**
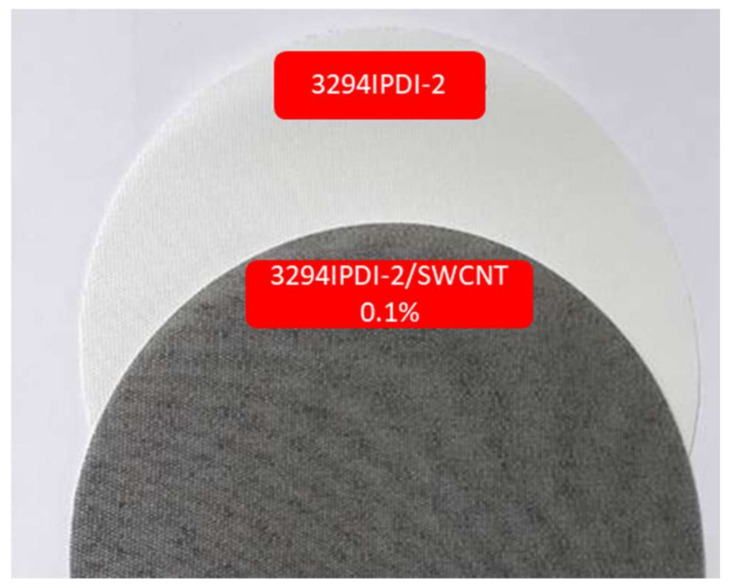
Appearance of UPRON fabric coated with: (**top**) 3294IPDI-2 and (**bottom**) 3294IPDI-2 dopped with 0.1 wt. % SWCNT.

From the results shown in Table 9 and Table 10, it can be concluded that the multifunctional effect, i.e., water column and antistatic properties, were achieved. From the values in Table 10 (electrostatic properties), it can be concluded that the addition of SWCNT in amount 0.05 wt. % to the WPUD is sufficient for the reliable antistatic effect complying with the standard EN 1149-5 for protective clothing. It is important to note that additivation with SWCNT is essential in ensuring that the fabrics pass the electrostatic properties standard for protective workwear. It must also be emphasized that doping with SWCNT does not substantially modify the water column properties of the coated UPRON fabric; however, the appearance (color) of the coated fabrics is altered, going from being transparent to grey-black (Figure 20). It is also important to mention that air permeability dramatically decreases and stiffness increases in all of the coated fabrics compared to untreated fabrics. This is logical when considering that we are applying a polymer layer to one of the faces of the fabric. Reduced air permeability can be a positive feature when it comes to outdoor sportwear with wind-stopper functionality. Regarding stiffness, sample 3294IPDI-2 provides the lowest stiffness values, which is consistent with the mechanical properties of this specimen, being the one with the lowest Young’s modulus and the highest Strain at break of the entire series. Finally, it is important to underline that all WPUD led to stable coating pastes with good runnability properties with the Werner–Mathis coating machine. Therefore, it is to be expected that the WPUD will lead to good results in later stages of industrial scaling.

**Figure 21:**
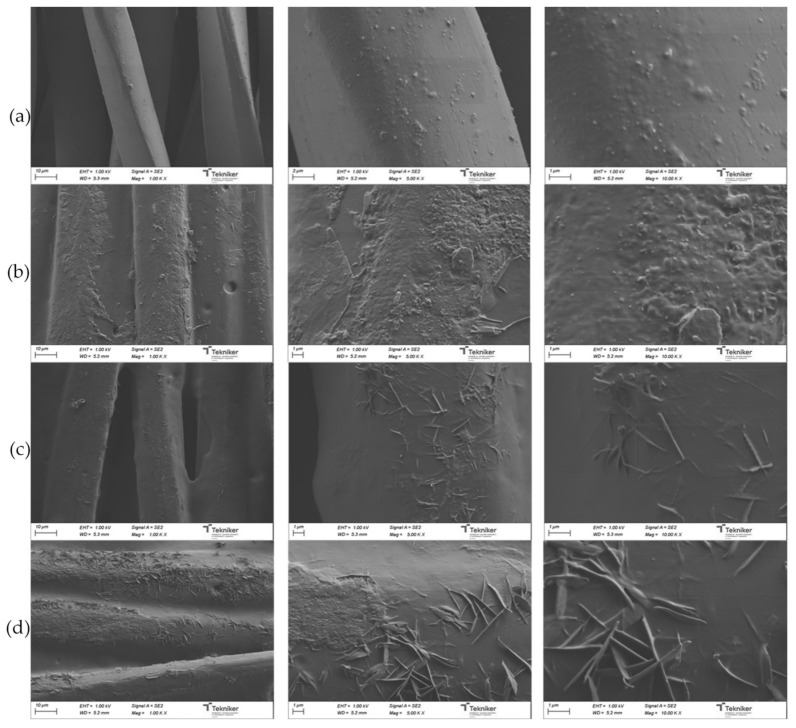
SEM micrographs for (**a**) uncoated UPRON fabric and UPRON fabrics coated with selected printing pastes: (**b**) 3294IPDI-2, (**c**) 3294IPDI-2 doped with 0.05 wt. % SWCNT, and (**d**) 3294IPDI-2 doped with 0.1 wt. % SWCNT.

Figure 21 shows the SEM micrographs of uncoated and coated UPRON fabrics with the printing pastes made from experiments 3294IPDI-2 and SWCNT-doped 3294IPDI-2. The presence of the coating in the fiber’s surface can be seen for all of the coated samples. The coatings are not distributed in a completely homogeneous way, as shown by the higher magnification images. The roughness of the surfaces seems to increase with the increase in SWCNT content in the 3294IPDI-2 matrix. In images with higher magnifications, aggregations of SWCNT are clearly observed. The SWCNT-doped coatings covered the fibers of the fabric, thus forming a three-dimensional network that contributes to the electrical conductivity of the textile. The low surface resistivity of the SWCNT coated textiles (see Table 10) is an indication of the proper distribution of SWCNT in the coatings [42]. As observed by other authors [43,44], the continuous structure of fibers in textiles is favorable in forming a conductive path. Moreover, some connections between the SWCNT can be observed.

## 4. Conclusions

In this work, the synthesis of a series of waterborne polyurethane-urea dispersions (WPUD) has been performed under strict sustainability and Eco-design criteria. For this purpose, the ternary mixture experimental design has been applied as a methodology to systematically explore different proportions between all of the building blocks employed in the synthesis, allowing us to establish a correlation between the structure of polymers and their properties. The WPUD obtained have a bio-based content ranging from 59 to 72%, and are free from tin catalysts and toxic solvents, like alkyl pyrrolidones, presenting a much more favorable carbon footprint and environmental impact than fluoropolymers, like PTFE or conventional petrol-based polyurethanes, which are commonly used in the textile sector as a membrane to confer hydrostatic pressure (water column) to technical-textiles. The obtained polyurethane-urea coatings offer excellent mechanical and thermal properties with elongations at break above 347.8%, good balance between elasticity and resilience and 10 wt. % loss measured by TGA above 276.5 °C for all of the WPUD series. The stability of the WPUD has been assessed by Z potential measurements and analytical centrifuge tests showing good stability after one year of storage, with no significant destabilization phenomena.

The application properties were also confirmed with good runnability of the coating pastes and proper performance, which, in some cases, equal and exceed those of conventional non-biobased polyurethanes. In this way, the technical feasibility of this type of polymer dispersions is revealed.

The design of experiments of ternary mixture used in this work has allowed for systematically exploring the ratio between the different building-blocks, revealing itself as a good methodology to explore the different characteristics of the designed polymers and establish a correlation between structure and properties that allows the fine-tuning of polymer properties as a function of the relationship between the different components of the mixture.

All of the synthesized polyurethane-ureas provide the coated substrates with remarkable water-repellence (WCA > 112°) and water column (≥26.9 cm), therefore being a more sustainable alternative than waterproof coatings based on fluoropolymers, such as PTFE. In addition to these valuable properties, the additivation of the polyurethane matrix with SWCNT has given rise to textile coatings with advanced functionalities, such as electrical conductivity, which are highly demanded in protective workwear and technical textiles. A surface resistivity as low as 3.6 × 106 Ω for polyester fabric coated with polymer matrix dopped with 0.1% SWCNT has been achieved, without a significant reduction in the static contact angle measurements or hydrostatic pressure, thus assessing the obtaining of multifunctional fabrics.

Undoubtedly, the synthesis of waterborne polymeric dispersions that were obtained from bio-based building-blocks, tin-free catalysts, and with low volatile organic content (VOC) is a very promising field of research that is gaining importance as the availability in the market of new bio-based building-blocks increases. Thus, it is foreseeable that, in the coming years, the textile-coating’s sector will benefit from the advances made in this field, being able to substitute fluoropolymers, like PTFE, or traditional petrol-based polyurethanes or polyacrylates by more sustainable alternatives, like the bio-based WPUD investigated in this work. Therefore, the fluorine-free coating agents based on waterborne dispersions of biobased polyurethane-ureas will contribute to lowering the environmental impact of water-proof finishing processes in the textile industry. In view of these promising results and the urgent need for replacement of fluoropolymers, which are known for its high impact and persistence in the environment, more efforts should be devoted to the design of new polymers based on fluorine-free building blocks that provide advanced barrier to fluids and multifunctional properties.

## Figures and Tables

**Table 1:** The overall simplex design table. Experiments that have been performed in this work are highlighted in grey.

Experiment	Matrix			Molar %			Hard Segment (wt. %)	Bio-Based Content (wt. %)
	DMBA	Priplast 3294	1,3-PDO	DMBA	Priplast 3294	1,3-PDO		
3294IPDI-1	1	0	0	60.00	40.00	0.00	40.6	59.4
3294IPDI-2	0	1	0	37.00	63.00	0.00	28.0	72.0
3294IPDI-3	0	0	1	37.00	40.00	23.00	38.8	62.6
4	1/2	1/2	0	48.50	51.50	0.00	33.4	66.6
5	1/2	0	1/2	48.50	40.00	11.50	39.7	61.0
6	0	1/2	1/2	37.00	51.50	11.50	32.6	68.0
3294IPDI-7	1/3	1/3	1/3	44.67	47.67	7.67	35.0	65.4
8	2/3	1/6	1/6	52.33	43.83	3.83	37.7	62.5
9	1/6	2/3	1/6	40.83	55.33	3.83	31.2	69.0
10	1/6	1/6	2/3	40.83	43.83	15.33	36.8	64.1

**Table 2:** Printing paste formulation employed to perform the coating of the fabrics with the WPUDs.

Composition	Printing Paste Formulation (g)
WPUD	85
water	15
Defoamer PR	1
Complex DG	2
DMEA	1
Thickener L-120	Added drop by drop under high shear until viscosity of 18,000 ± 50 cPs is reached (Brookfield RV 6/30)
Dry polyurethane-urea content	30 ± 1%

**Table 3:** Standards employed to evaluate the properties of the coated textiles.

Property	Standard
Determination of resistance to water penetration-Hydrostatic pressure test (water column)	EN 20811:1992
Fabric stiffness	ČSN 80 0858
Determination of the permeability of fabrics to air	ISO 9237:1995

**Table 4:** The standards employed to evaluate electrostatic properties of the coated textiles and minimum compliance values.

Electrostatic Properties	Standard	Minimum Compliance Values
Surface resistivity & Specific surface resistivity	EN 1149-1	Surface resistance ≤ 2.5 × 10^9^
Vertical resistance	EN 1149-2	Electrical resistance > 10^5^
Charge decay (inductive charge)	EN 1149-3, method 2	t_50_ < 4 s or S > 0.2 values
Electrostatic properties. Performance requirements and material design.	EN 1149-5	t50 < 4 s or S > 0.2 values; or surface resistance is ≤ 2.5·10^9^

**Table 5:** Thermal properties of WPUD films evaluated by DSC, TGA, and DMTA.

Reference	DSC ^a^	TGA ^b^				DMTA ^c^
	*T*_g_ (°C)	*T*_10%_ (°C)	*T*_dS1_ (°C)	*T*_dS2_ (°C)	*T*_dS3_ (°C)	*T*_α_ (°C)
3294IPDI-1	−53.8	276.5	254.9	326.1	423.2	109.2
3294IPDI-2	−50.8	308.6	263.3	327.2	420.6	68.9
3294IPDI-3	−53.7	297.6	261.1	326.8	422.5	123.5
3294IPDI-7	−51.2	300.1	261.8	326.2	422.7	106.2
Priplast 3294	−66.0	402.7	-	-	414.4	N.D. ^d^

^a^*T*_g_ values determined by DDSC. ^b^ TGA characterization of the WPUD films: *T*_10%_ is the temperature at which a 10 wt. % loss was observed in the TGA traces recorded at 10 °C min^−1^_;_ *T*_dS1_, *T*_dS2_, *T*_dS3_ are the temperatures of maximum degradation rate for first, second a third degradation stages, respectively. ^c^ *T*_α_, relaxation temperature calculated from the maximum value of tan δ by DMTA. ^d^ Data not given on Priplast 3294 due to not appropriate state for DMTA (melts).

**Table 6:** Mechanical characterization of WPUD and RD27 films.

Reference	E ^a^ (Mpa)	σ_100%_^a^ (Mpa)	σ_b_^a^ (Mpa)	ε_b_^a^ (%)
3294IPDI-1	120.6 ± 4.0	12.5 ± 0.1	35.9 ± 1.2	415.4 ± 11.4
3294IPDI-2	25.1 ± 0.3	5.4 ± 0.0	35.4 ± 1.4	625.4 ± 15.8
3294IPDI-3	161.0 ± 9.3	11.7 ± 0.2	28.4 ± 2.0	347.8 ± 11.9
3294IPDI-7	103.9 ± 6.1	9.1 ± 0.1	28.7 ± 1.3	427.3 ± 8.3
3294IPDI-2/ SWCNT 0.1 wt. %^b^	19.3 ± 1.2	4.8 ± 0.2	25.8 ± 6.1	735.0 ± 6.1
RD27 ^b^	35.4 ± 0.4	5.6 ± 0.0	33.4 ± 4.7	700.7 ± 80.5

E ^a^: Young modulus, σ_100%_: stress at 100% strain, σ_b_: stress at break, ε_b_: strain at break. ^b^ Non-biobased commercial polyurethane (RD27) and 3294IPDI-2 WPUD additivated with 0.1 wt. % SWCNT (3294IPDI-2/SWCNT 0.1 wt. %) have been included for comparison.

**Table 7:** A summary of the particle size and PdI measurements, average particle size, and Z-potential values for synthesized WPUD dispersions.

Reference	pH at 35% of Solid Content	Average Particle Size (nm)	PdI	Z Potential (mV)
3294IPDI-1	8.1	50 ± 7	0.12 ± 0.1	−40.5 ± 6.0
3294IPDI-2	8.2	217 ± 40	0.11 ± 0.1	−38.7 ± 7.9
3294IPDI-3	8.1	49 ± 10	0.20 ± 0.1	−39.8 ± 4.3
3294IPDI-7	8.0	48 ± 15	0.19 ± 0.1	−43.5 ± 4.7

**Table 8:** WCA, θ_Av_, θ_Re_, and calculated CAH of glass and UPRON substrates coated with WPUD and RD27.

Coating Reference	Substrate							
	Glass Slides				UPRON ^a^			
	WCA (°)	θ_Av_ (°)	θ_Re_ (°)	CAH^b^ (°)	WCA (°)	θ_Av_ (°)	θ_Re_ (°)	CAH ^b^ (°)
Uncoated	spreads	-	-	-	wets	-	-	-
3294IPDI-1	98.4 ± 1.3	97.0 ± 3.3	94.8 ± 0.8	2.3 ± 3.4	121.4 ± 1.8	122.7 ± 3.8	119.4 ± 5.1	3.2 ± 1.5
3294IPDI-2	86.0 ± 1.9	91.5 ± 4.5	84.4 ± 5.6	7.1 ± 6.5	119.7 ± 4.0	125.1 ± 4.4	121.6 ± 5.7	2.4 ± 2.2
3294IPDI-3	92.4 ± 2.7	92.9 ± 1.0	87.1 ± 4.5	5.9 ± 4.6	116.3 ± 2.8	117.1 ± 1.1	113.8 ± 1.1	3.3 ± 1.0
3294IPDI-7	86.9 ± 0.6	90.4 ± 2.2	88.2 ± 2.2	2.2 ± 3.1	113.2 ± 7.0	115.3 ± 6.4	111.8 ± 5.7	3.5 ± 1.0
RD27	78.2 ± 1.0	79.4 ± 1.0	77.0 ± 1.3	2.4 ± 1.6	106.8 ± 2.4	111.2 ± 0.4	110.6 ± 0.7	0.6 ± 1.0
3294IPDI-2/ SWCNT 0.05 wt. %	78.3 ± 1.2	90.8 ± 3.6	86.2 ± 1.2	4.6 ± 3.8	111.7 ± 1.4	112.7 ± 3.8	109.4 ± 2.8	3.3 ± 3.2
3294IPDI-2/ SWCNT 0.1 wt. %	89.2 ± 1.6	96.0 ± 3.0	94.3 ± 2.7	1.6 ± 4.1	114.9 ± 5.0	120.5 ± 1.4	115.7 ± 1.6	4.8 ± 0.9

^a^ The Dry add-on of the coated UPRON fabrics was 25 ± 2 g m^−2^ for all the coated samples. ^b^ CAH = θ_Av_ − θ_Re_ (°).

**Table 9:** Stiffness, water column, and air permeability of UPRON fabrics uncoated and coated by knife coating procedure.

Coating Reference	Dry Add-on (g/m2)	Stiffness [mN]		Water Column (cm)	Air Permeability (mm/s)
		Warp	Weft		
Uncoated	-	17.4	9.7	<15 soaked	85.3
3294IPDI-1	23.5	153.0	50.3	26.9	3.7
3294IPDI-2	27.0	127.0	42.2	38.0	0.7
3294IPDI-3	24.9	154.0	50.3	28.9	2.6
3294IPDI-7	26.0	161.0	47.3	29.3	1.8
RD27	22.0	155.0	43.9	34.0	0.4
3294IPDI-2/SWCNT 0.05 wt. %	24.1	124.0	38.9	32.7	4.4
3294IPDI-2/SWCNT 0.1 wt. %	24.0	87.0	31.9	33.8	3.6

**Table 10:** Summary of electrostatic properties of UPRON fabrics coated with 3294IPDI-2 and 3294IPDI-2 doped with 0.05 and 0.1 wt. % SWCNT.

Coating Reference	Electrostatic Property	Unit	Limit	Value	Requirements EN 1149-5
3294IPDI-2	Surface resistivity	Ω	≤2.5 × 10^9^	9.2 × 10^12^	FAIL
	Charge decay (inductive charging)	s	t_50_ < 4	>30	
		-	S > 0.2	0.03	
	Vertical resistance	Ω	>10^5^	1.1.10^12^	
3294IPDI-2/SWCNT 0.05 wt. %	Surface resistivity	Ω	≤2.5 × 10^9^	3.8 × 10^6^	PASS
	Charge decay (inductive charging)	s	t_50_ < 4	<0.01	
		-	S > 0.2	0.99	
	Vertical resistance	Ω	>10^5^	2.8 × 10^11^	
3294IPDI-2/SWCNT 0.1 wt. %	Surface resistivity	Ω	≤2.5 × 10^9^	3.6 × 10^6^	PASS
	Charge decay (inductive charging)	s	t_50_ < 4	<0.01	
		-	S > 0.2	0.99	
	Vertical resistance	Ω	>10^5^	3.2 × 10^11^	

## Data Availability

The raw/processed data required to reproduce these findings cannot be shared at this time as the data also forms part of an ongoing study.

## Supplementary Materials

The following are available online at https://www.mdpi.com/article/10.3390/polym13101624/s1, Figure S1: 1H NMR spectra of starting polyol Priplast 3294 and all the synthesized WPUD, Figure S2: 1H NMR spectra of model compound IPDI:DMBA (molar ratio 2:1), Figure S3: 1H NMR spectra of model compound IPDI:PDO (molar ratio 2:1), Figure S4: 1H NMR spectra of model compound IPDI:Priplast 3294 (molar ratio 2:1), Figure S5: 1H NMR spectra of model compound IPDI:EDA (molar ratio 2:1), Figure S6: 1H NMR spectra of model compound IPDI:EtOH (molar ratio 1:3), Figure S7: DSC of Priplast 3294 were Tm, ΔHm and Tc, ΔHc have been determined during second heating and cooling, respectively, Figure S8: TGA curves of starting polyol Priplast 3294, commercial non-biobased polyurethane RD27 (polyether polyurethane) and WPUD 3294IPDI-7. Mass losses (thick lines) and derivative curves (dashed lines), Figure S9: Left, equipment used to perform stress–strain measurements. Center, dimensions of the dumbbell type 4 specimens used in this test. Right, generic stress–strain graph with characteristic parameters (elastic modulus, stress at break, strain at break), Figure S10: Particle size distribution measured by DLS, Figure S11: Transmission profiles of the synthesized WPUD dispersions subjected to 2000× *g* and 40 °C centrifuge tests using a path length of 10 mm, Figure S12: Transmission profiles of the synthesized WPUD dispersions subjected to 2000× *g* and 4 °C centrifuge test using a path length of 10 mm.
