# Supplementary material for: Biobased Waterborne Polyurethane-Urea/SWCNT Nanocomposites for Hydrophobic and Electrically Conductive Textile Coatings
Source: Polymers (Basel). 2021 May 17;13(10):1624. doi: 10.3390/polym13101624 (PMC8156085; doi:10.3390/polym13101624)
Supplement: Supplementary file 1 [file polymers-13-01624-s001.zip › polymers-1220692-supplementary.pdf]

SUPPORTING INFORMATION

Amado Lacruz [1,2,*], Mireia Salvador [1], Miren Blanco [3], Karmele Vidal [3], Amaia M. Goitandia [3], Lenka Martinková [4], Martin Kyselka [4], and Antxon Martínez de Ilarduya [2]

[1] Color Center, S.A. Ptge. Marie Curie 3, Nau 6, 08223 Terrassa, Spain; alacruz@colorcenter.es (A.L.); msalvador@colorcenter.es (M.S.)

[2] Departament d'Enginyeria Química, Universitat Politècnica de Catalunya, ETSEIB, Diagonal 647,
08028 Barcelona, Spain; antxon.martinez.de.ilarduia@upc.edu (A.M.d.I.)

[3] Tekniker, Basque Research and Technology Alliance (BRTA), Surface Chemistry and Nanotechnology Unit, Iñaki Goenaga 5, 20600 Gipuzkoa, Spain; miren.blanco@tekniker.es (M.B.); karmele.vidal@tekniker.es (K.V.); amaia.martinez@tekniker.es (A.M.G.)

[4] Inotex spol. s r.o, Stefanikova 1208, 54401 Dvur Kralove n.L., Czech Republic; martinkova@inotex.cz (L.M.);
kyselka@inotex.cz (M.K.)

* Correspondence: Amado Lacruz (amado.lacruz@upc.edu). Phone number: +34 937861113

Table of Contents

1 NMR S2
2 DSC S6
3 TGA S6
4 MECHANICAL PROPERTIES S7
5 PSD S7
6 ANALYTICAL CENTRIFUGUE S8

1 NMR

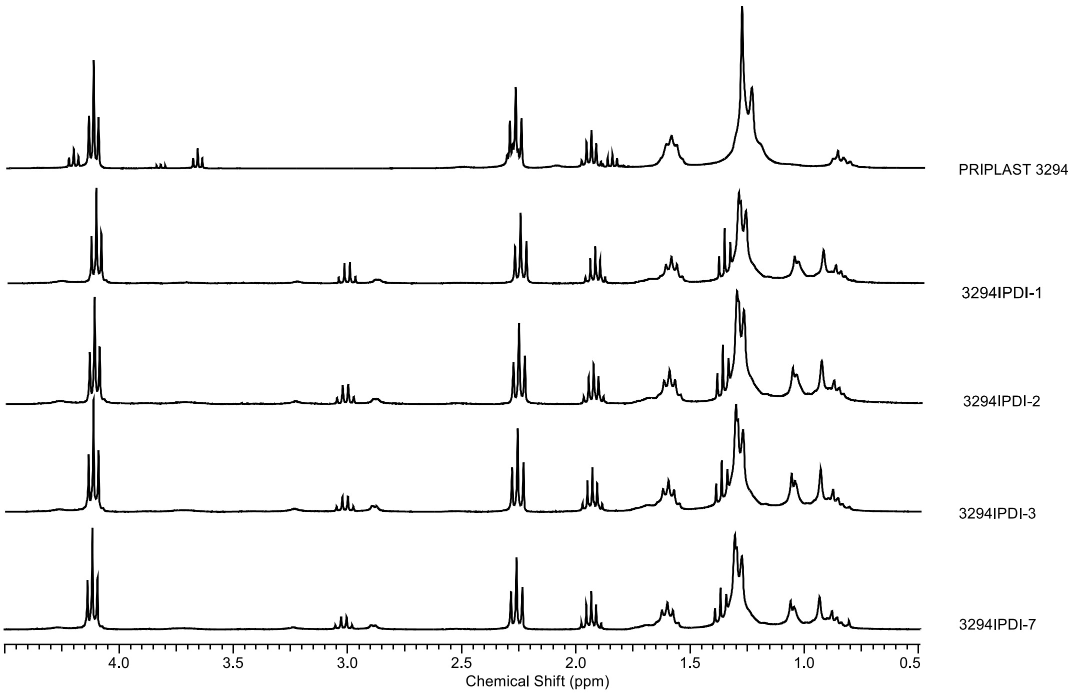

Figure S1. ^{1}H NMR spectra of starting polyol Priplast 3294 and all the synthesized WPUD.

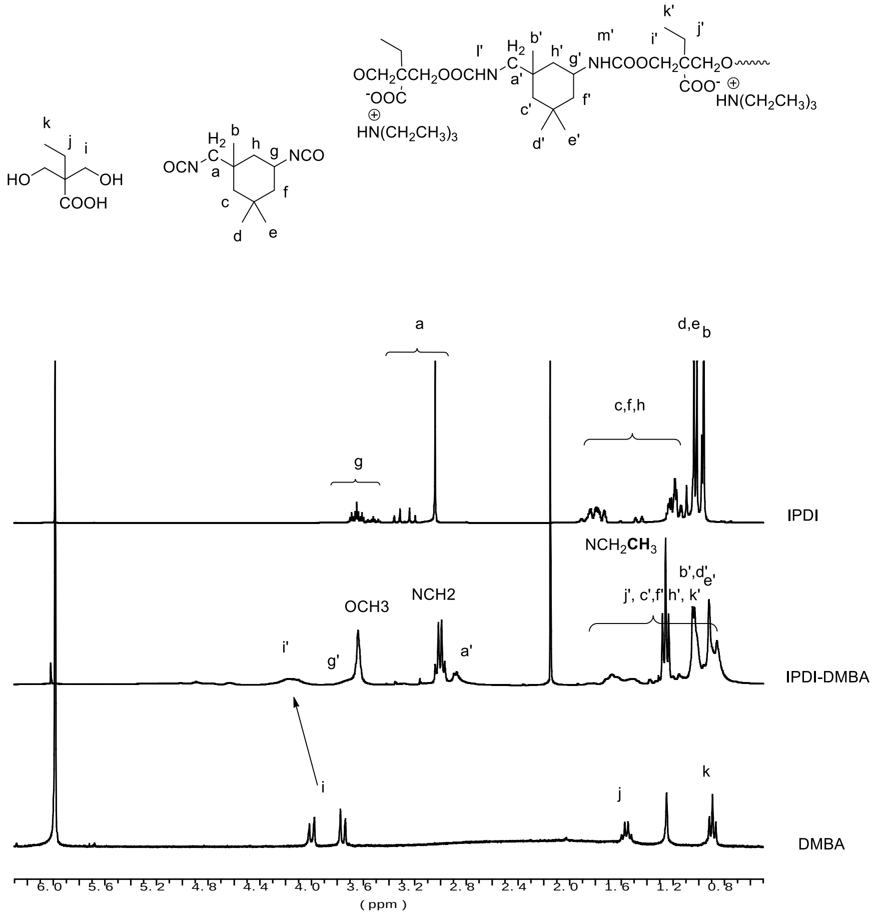

Figure S2. ¹H NMR spectra of model compound IPDI:DMBA (molar ratio 2:1).

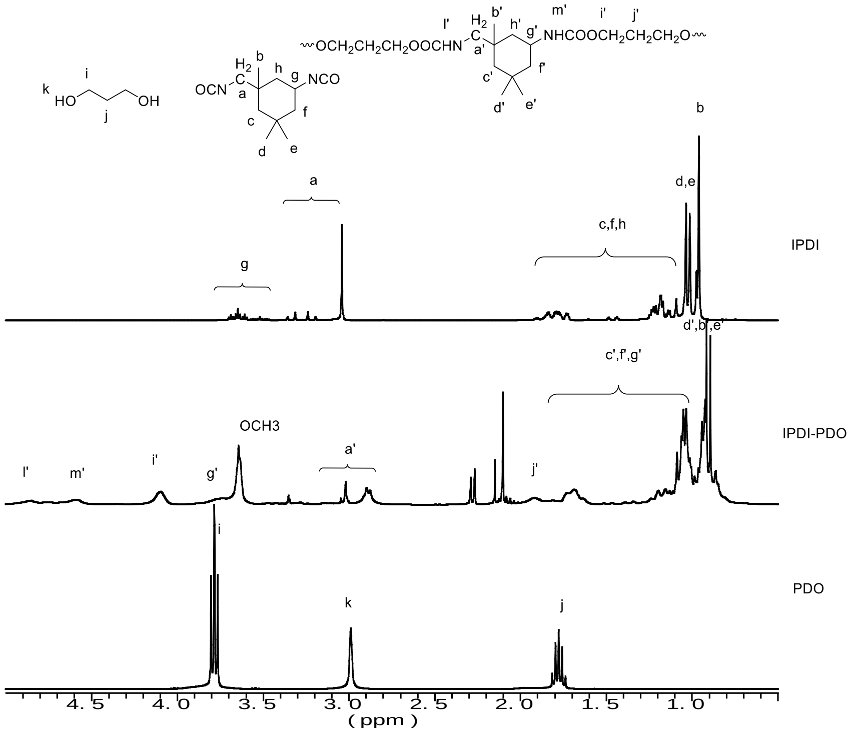

Figure S3. ¹H NMR spectra of model compound IPDI:PDO (molar ratio 2:1).

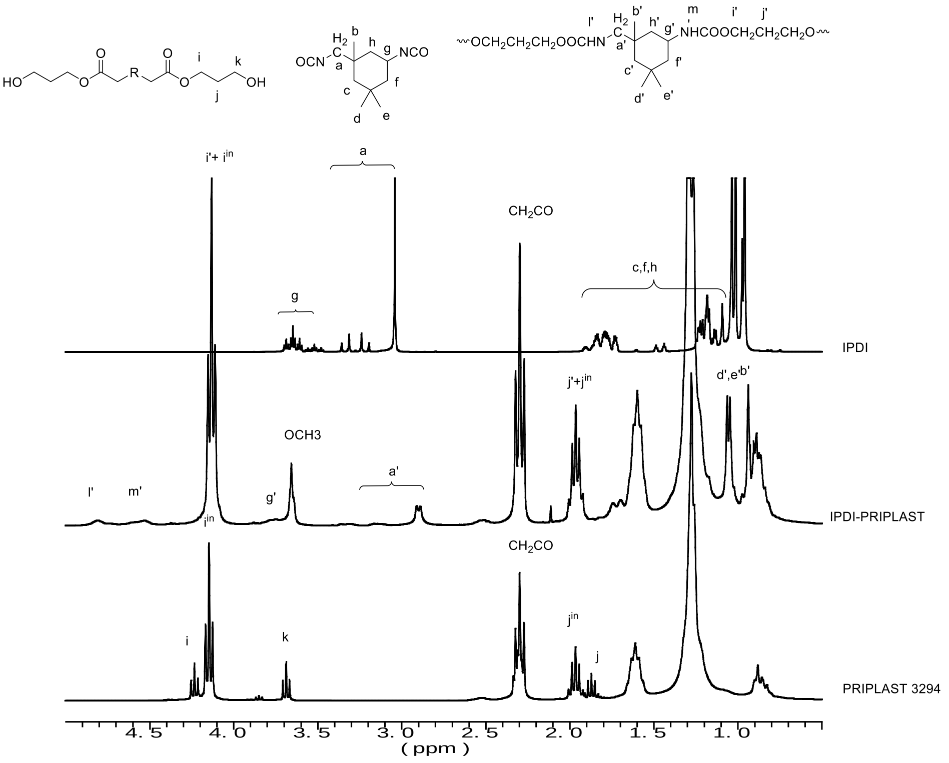

Figure S4. ^{1}H NMR spectra of model compound IPDI:Priplast 3294 (molar ratio 2:1).

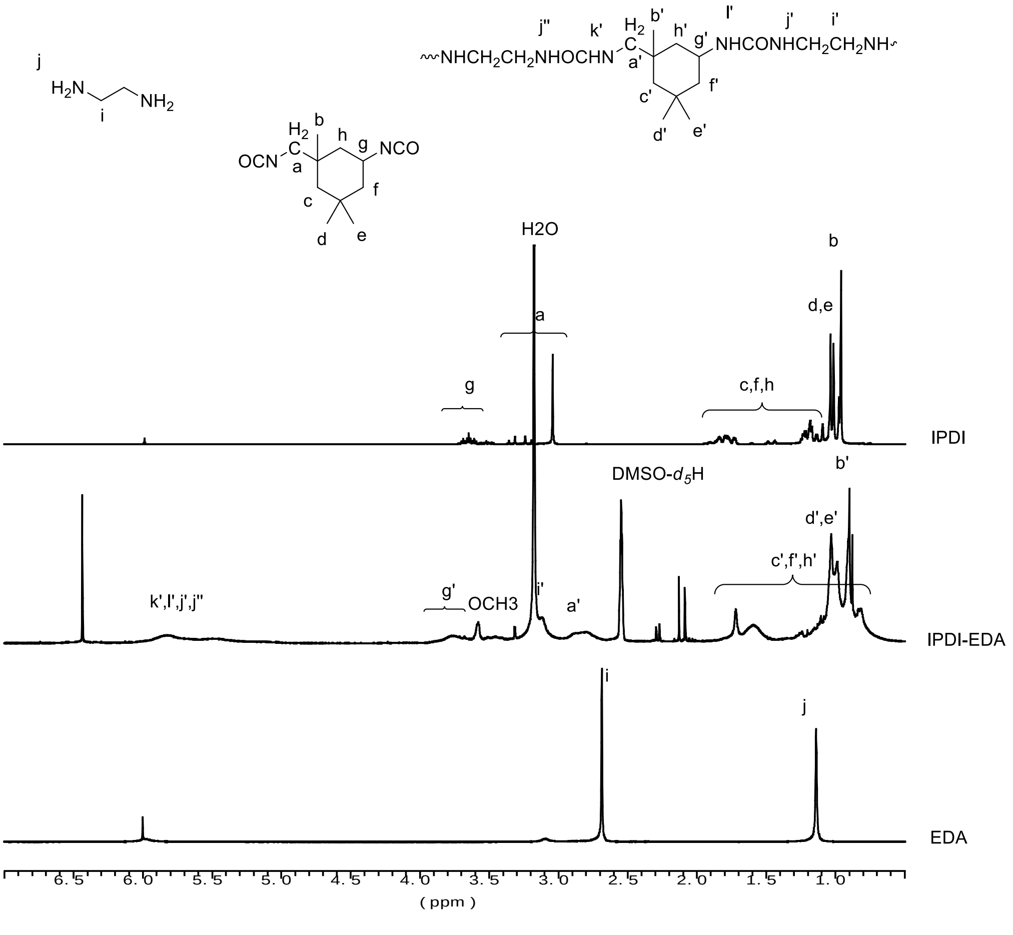

Figure S5. ^{1}H NMR spectra of model compound IPDI:EDA (molar ratio 2:1).

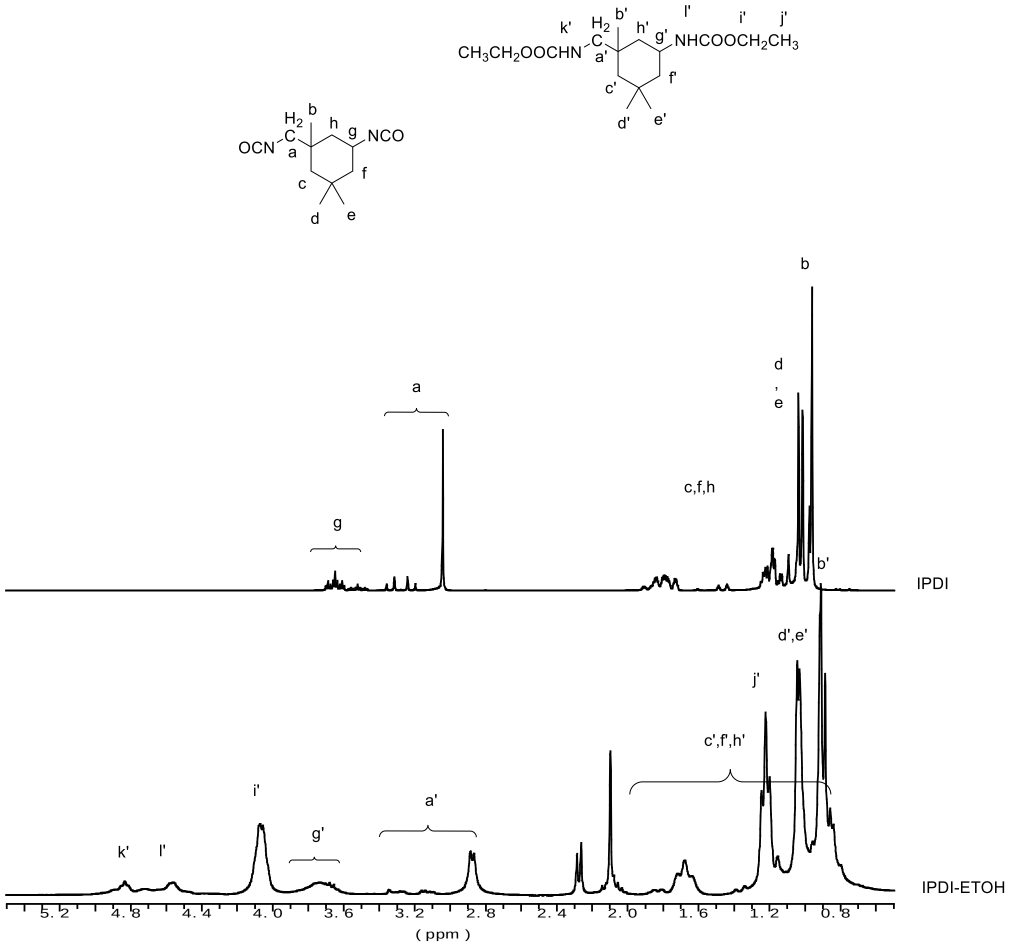

Figure S6. ^{1}H NMR spectra of model compound IPDI:EtOH (molar ratio 1:3).

2 DSC

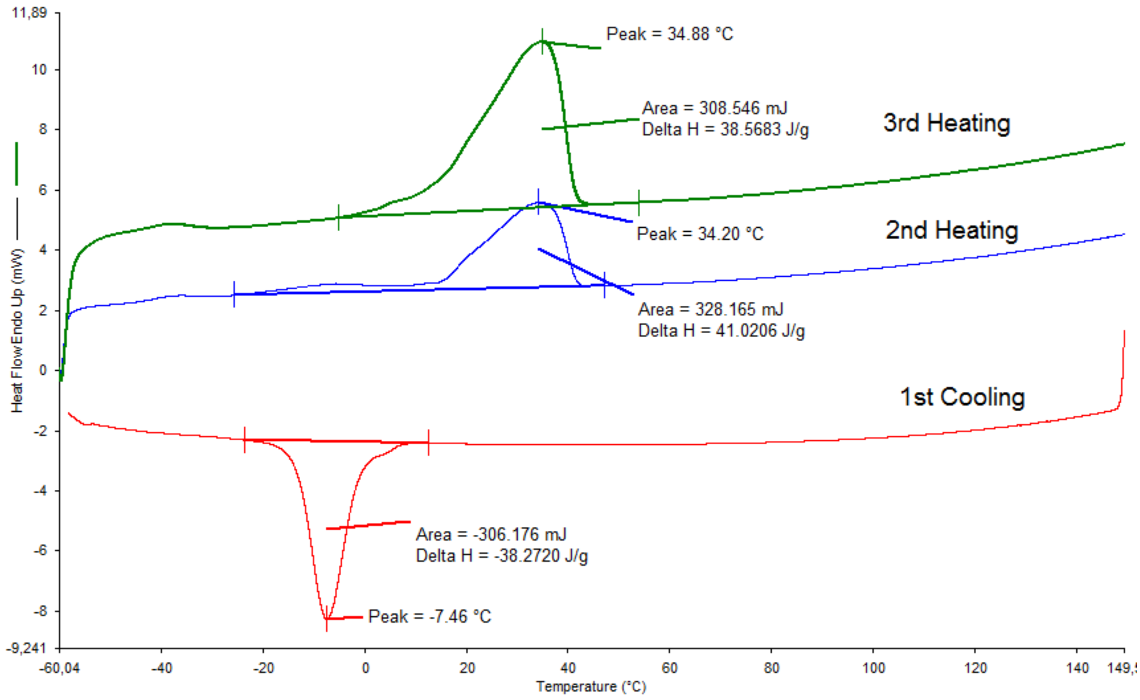

Figure S7. DSC of Priplast 3294 were T_m, ΔH_m and T_c, ΔH_c have been determined during second heating and cooling, respectively.

3 TGA

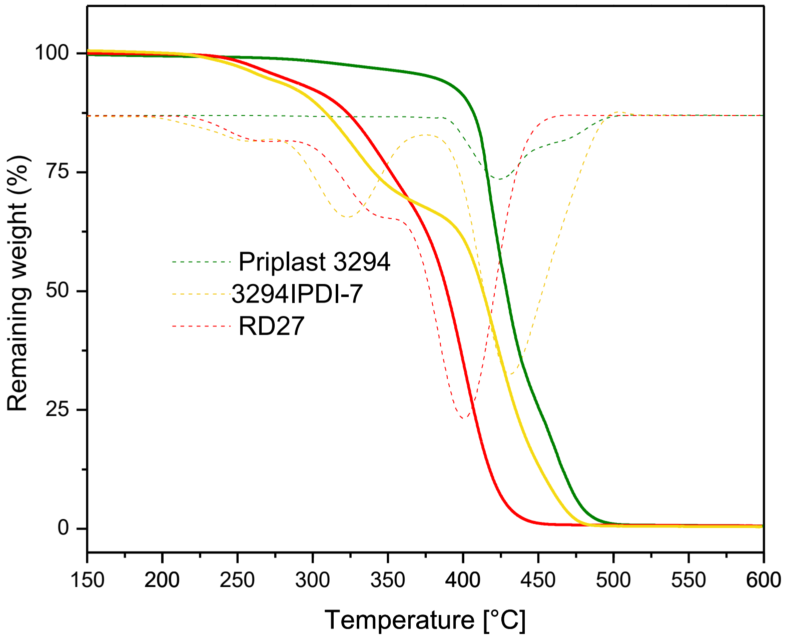

Figure S8. TGA curves of starting polyol Priplast 3294, commercial non-biobased polyurethane RD27 (polyether polyurethane) and WPUD 3294IPDI-7. Mass losses (thick lines) and derivative curves (dashed lines).

4 MECHANICAL PROPERTIES

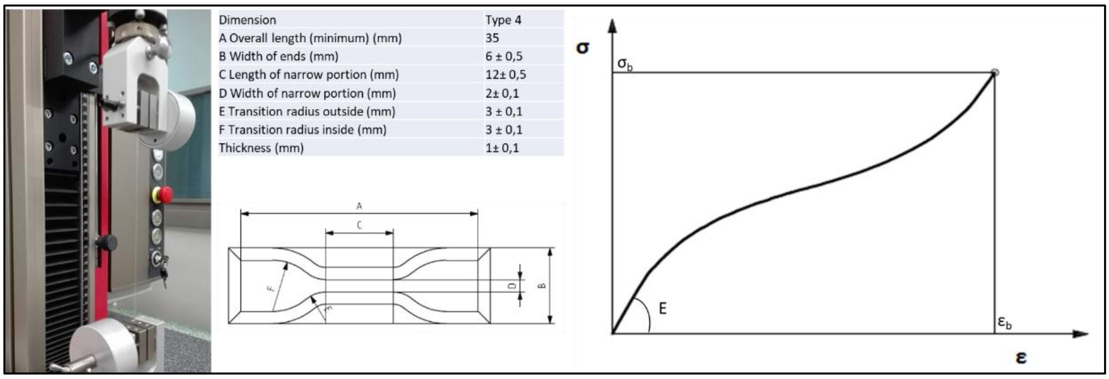

Figure S9. Left, equipment used to perform stress-strain measurements. Center, dimensions of the dumbbell type 4 specimens used in this test. Right, generic stress-strain graph with characteristic parameters (elastic modulus, stress at break, strain at break).

5 PSD

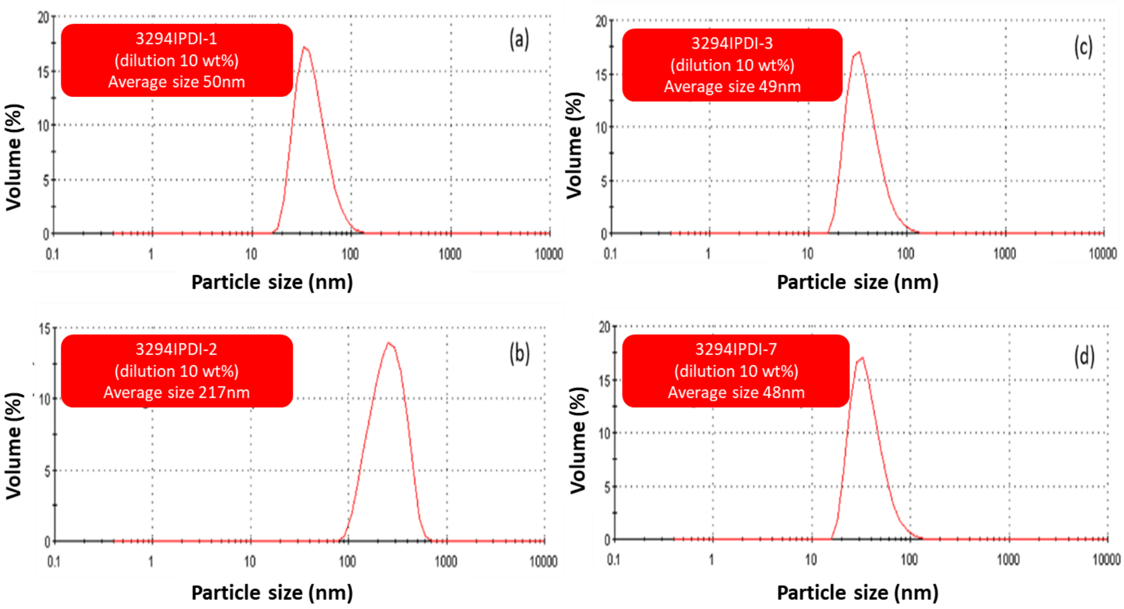

Figure S10. Particle size distribution measured by DLS.

6 ANALYTICAL CENTRIFUGE

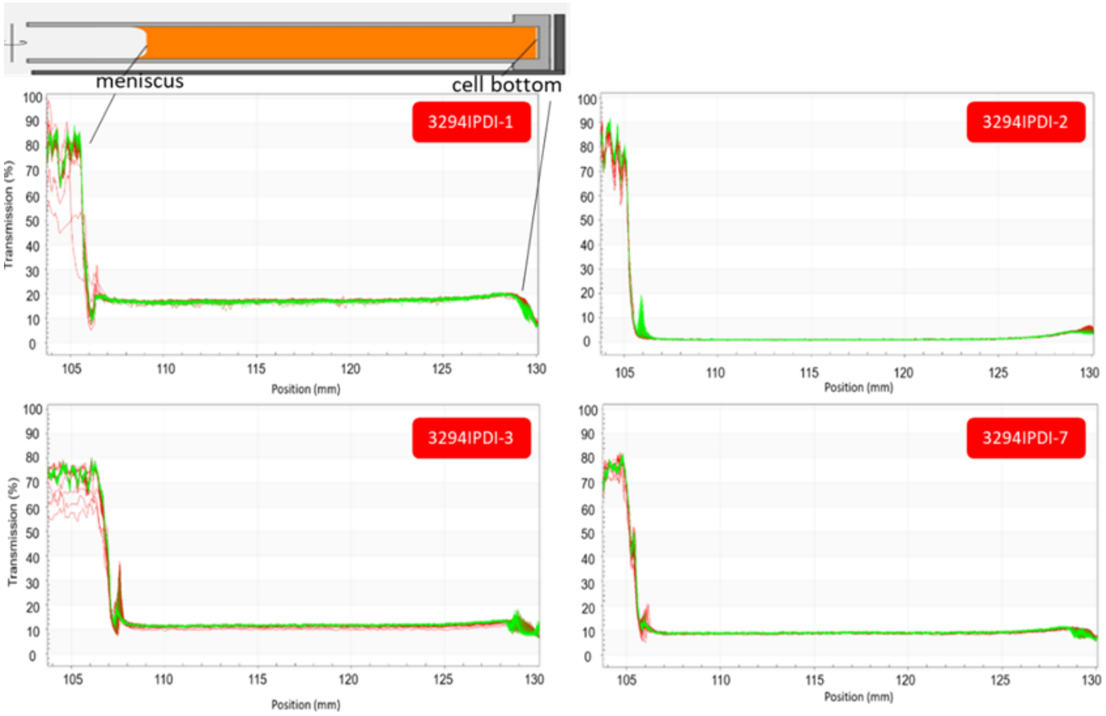

Figure S11. Transmission profiles of the synthesized WPUD dispersions subjected to 2000 g and 40 ºC centrifuge tests using a path length of 10 mm.

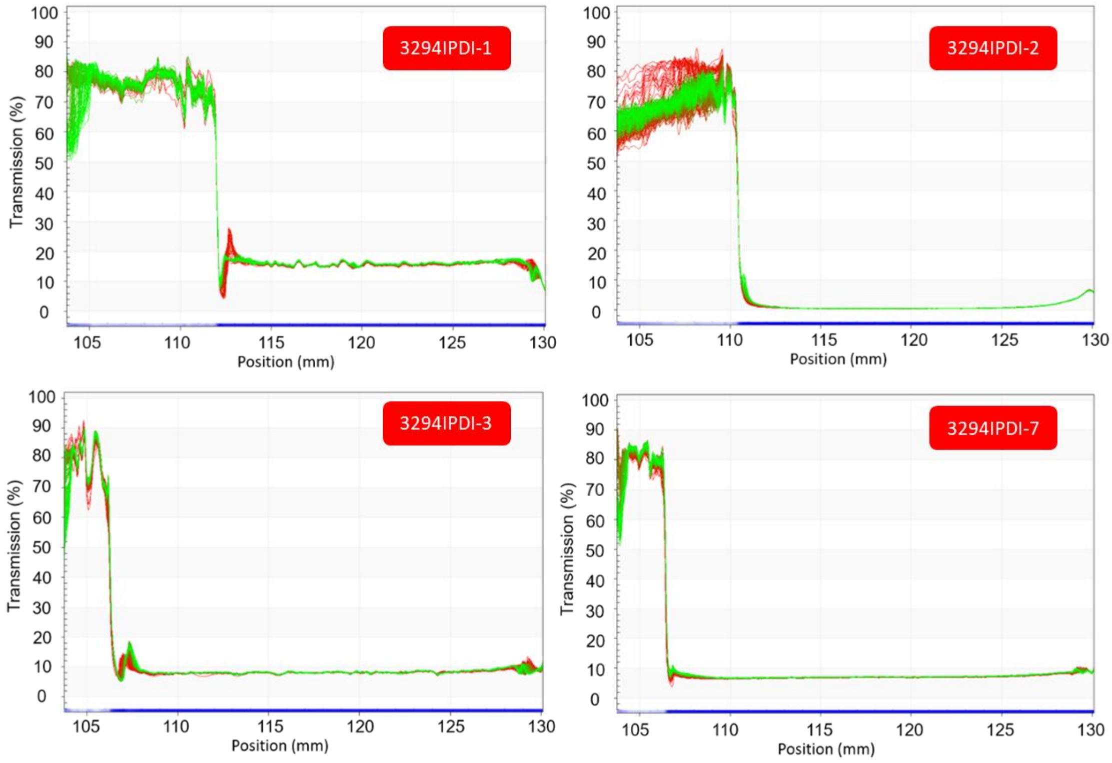

Figure S12. Transmission profiles of the synthesized WPUD dispersions subjected to 2000 g and 4 ºC centrifuge test using a path length of 10 mm.
